# Application of Rotating Magnetic Fields Increase the Activity of Antimicrobials Against Wound Biofilm Pathogens

**DOI:** 10.1038/s41598-017-18557-7

**Published:** 2018-01-09

**Authors:** A. F. Junka, R. Rakoczy, P. Szymczyk, M. Bartoszewicz, P. P. Sedghizadeh, K. Fijałkowski

**Affiliations:** 1 Department of Pharmaceutical Microbiology and Parasitology, Wrocław Medical University, Borowska 211A, 50-556 Wrocław, Poland; 2 Institute of Chemical Engineering and Environmental Protection Processes, Faculty of Chemical Technology and Engineering, West Pomeranian University of Technology, Szczecin, Piastów 42, 71-065 Szczecin, Poland; 3 Centre for Advanced Manufacturing Technologies (CAMT/FPC), Faculty of Mechanical Engineering, Wrocław University of Science and Technology, Łukasiewicza 5, 50-371 Wrocław, Poland; 4 Center for Biofilms, Ostrow School of Dentistry of University of Southern California, 925 West 34th, Los Angeles, California, United States of America; 5 Department of Immunology, Microbiology and Physiological Chemistry, Faculty of Biotechnology and Animal Husbandry, West Pomeranian University of Technology, Szczecin, Piastów 45, 70-311 Szczecin, Poland

## Abstract

Infective complications are a major factor contributing to wound chronicity and can be associated with significant morbidity or mortality. Wound bacteria are protected in biofilm communities and are highly resistant to immune system components and to antimicrobials used in wound therapy. There is an urgent medical need to more effectively eradicate wound biofilm pathogens. In the present work, we tested the impact of such commonly used antibiotics and antiseptics as gentamycin, ciprofloxacin, octenidine, chlorhexidine, polihexanidine, and ethacridine lactate delivered to *Staphylococcus aureus* and *Pseudomonas aeruginosa* biofilms in the presence of rotating magnetic fields (RMFs) of 10–50 Hz frequency and produced by a customized RMF generator. Fifty percent greater reduction in biofilm growth and biomass was observed after exposure to RMF as compared to biofilms not exposed to RMF. Our results suggest that RMF as an adjunct to antiseptic wound care can significantly improve antibiofilm activity, which has important translational potential for clinical applications.

## Introduction

Wounds, especially chronic ones and protracted with infective complications, become an increasing burden for patients and healthcare systems and lead to significant deterioration of life or, if untreated appropriately, to death^1^. The infective complication of wound ulcerations of virtually each etiology: venous, arterial, diabetic, neoplastic, bedsores, or of burn origin impedes or stops the process of wound healing and is responsible for persistence or chronicity of a wound. In the past, such infections were treated locally by means of antibiotic-containing creams or ointments. However, local application of antibiotics has contributed to rise of antibiotic-resistant pathogens. Due to this and other disadvantages related to the use of locally administered antibiotics, they are no longer indicated for chronic wound care^2^.

In cases of infected wounds, systemic antibiotic therapy is now recommended and serves to prevent bacterial penetration from the wound bed to deeper tissues including hematogenous spread^3^. Thus, in local wound treatment, options include debridement (of surgical, biological or chemical nature), intravenous antibiotic therapy, and antiseptic application. Antiseptic application has become progressively common in wound therapy^4^. Antiseptics are locally administered antimicrobials, and due to non-specific mechanisms of action, the use of a majority of them does not lead to microbial resistance. There are two major mechanisms of action for antiseptics. The first involves charged molecules in the antiseptic binding to bacterial cell walls and membranes, resulting in destruction of these structures. This is followed by leakage of cytoplasmic components into the environment, enzymatic malfunctions, and to bacterial cell death inevitably. Such a phenomenon is seen with one of the most potent antiseptics, octenidine dihydrochloride (Octenidine, OCT), and with other major antiseptics such as chlorhexidine (CHX) and polihexanidine (PHMB)^5^. The second mechanism for antiseptic activity relies on the ability to destroy and denature bacterial proteins. The most common antiseptic displaying such a mechanism of action is povidone-iodine (PVP-I)^5^. Other mechanisms include binding and despairing of microbial DNA, as with the commonly used antiseptic ethacridine lactate^6^. However, use of an antiseptic below a minimum inhibitory concentration can contribute to a rise in microbial cross-resistance to both antiseptics and antibiotics because of the survival of resistant cells within a population. This clinically dangerous phenomenon is particularly well-described for Gram-negative pathogens such as *Pseudomonas aeruginosa* and *Acinetobacter* spp. and it is related to the overexpression of efflux pumps able to actively remove chlorhexidine and antibiotics outside of the bacterial cytoplasm^7–9^.

Although modern antiseptics are very efficient antimicrobials, bacteria living within chronic wounds are able to survive in many cases. One of the reasons for this is the ability of microbes to form a biofilm, e.g. a sessile or bound community of cells embedded within polymeric matrix that gives them adaptive tolerance to immunity and imparts antimicrobial resistance^10^. The wound environment itself is the second reason, explaining many cases of failed therapy. First, the majority of chronic wounds produce an exudate, a type of turbid cellular fluid which can dilute an antiseptic. Second, various irregularities and niches may be present in the wound topography. These niches are used by microbes as a surface to replicate and as shelter from unfavorable agents^11^. Third, in the course of their evolutionary relationship with humans, predominant wound pathogens developed the ability to use host fibrinogen and to transform it into fibrin. These fibrin accretions form a clot with bacteria contained within it, protecting them from antimicrobials and immune responses^12^.

At first glance, increasing the concentration of active substances in an antiseptic may appear to be a possible solution for overcoming the aforementioned issues. Unfortunately, increases of antiseptic concentrations can correlate with elevated levels of cytotoxicity that leads to host cell damage^6^. Therefore, we asked the following question: how can we increase antiseptic/antibiotic efficacy without increasing concentrations? We hypothesized that the application of rotating magnetic fields (RMF) coupled with antimicrobials could be a possible solution to this issue.

Magnetic fields in general are part of an electromagnetic field, or physical field produced by electrically charged objects. There are numerous scientific reports on the effects, both negative and positive ones, concerning the impact of magnetic fields on humans and on other organisms. The negative effects include carcinogenesis mainly^13^, while positive ones include stimulation of bone repair and chronic wound healing, or even such puzzling effects as attenuation of anxiety behavior in rats^14–16^. Most magnetic field effects are frequency and exposure-time related. The biological background of these phenomena still need to be elucidated. Proposed explanations focus mainly on the impact of magnetic fields on charged molecules, with a special emphasis placed on various cell membrane structures and electrically charged molecules in bacterial environments (e.g. culture media, water, or other liquids within which microbes are suspended)^17,18^.

With respect to microbiology and unicellular microorganisms, biological effects of magnetic fields include stimulation or inhibition, depending on the microorganisms involved, magnetic field frequency or time of exposure in addition to environmental factors. There is ongoing debate between various research teams concerning the nature and true impact of magnetic fields on bacteria and fungi^19^. There is even less data concerning RMF – a field where opposite poles rotate around a central point or axis. Our research team has long-standing experience in studying RMF applications, and we previously demonstrated the impact of RMF on growth, metabolic activity, and biofilm formation of *Staphylococcus aureus*, *Escherichia coli*, *Acinetobacter baumanii*, *Pseudomonas aeruginosa*, *Serratia marcescens*, *Streptococcus mutans*, *Cronobacter sakazakii*, *Klebsiella oxytoca* and *Staphylococcus xylosus* ^20^. Moreover, in a distinct line of investigation we demonstrated that RMF-induced bacterial growth alteration may be used for production of bacterial cellulose (BC) displaying more favorable properties than BC produced by bacteria in non-RMF-induced environments^21^. Since BC is used for manufacturing of high-quality dressings for chronic wounds, we identified that it was important to investigate RMF coupled with antiseptics and antibiotics against chronic wound pathogens. We hypothesized that RMF could have an impact on antimicrobial activity and penetrability, resulting in greater efficacy for eradicating bacteria. This assumption is backed up by results of other experiments, where other types of electromagnetic fields were used to disturb microbial structures and led to permanent damage to cell membranes, presumably by membrane irreversible electroporation^22,23^.

Thus, the aim of the research presented herein was to investigate different variants of RMF exposure combined with antimicrobial application against common wound pathogens, namely *S. aureus* and *P. aeruginosa*.

## Results

**Figure 1:**
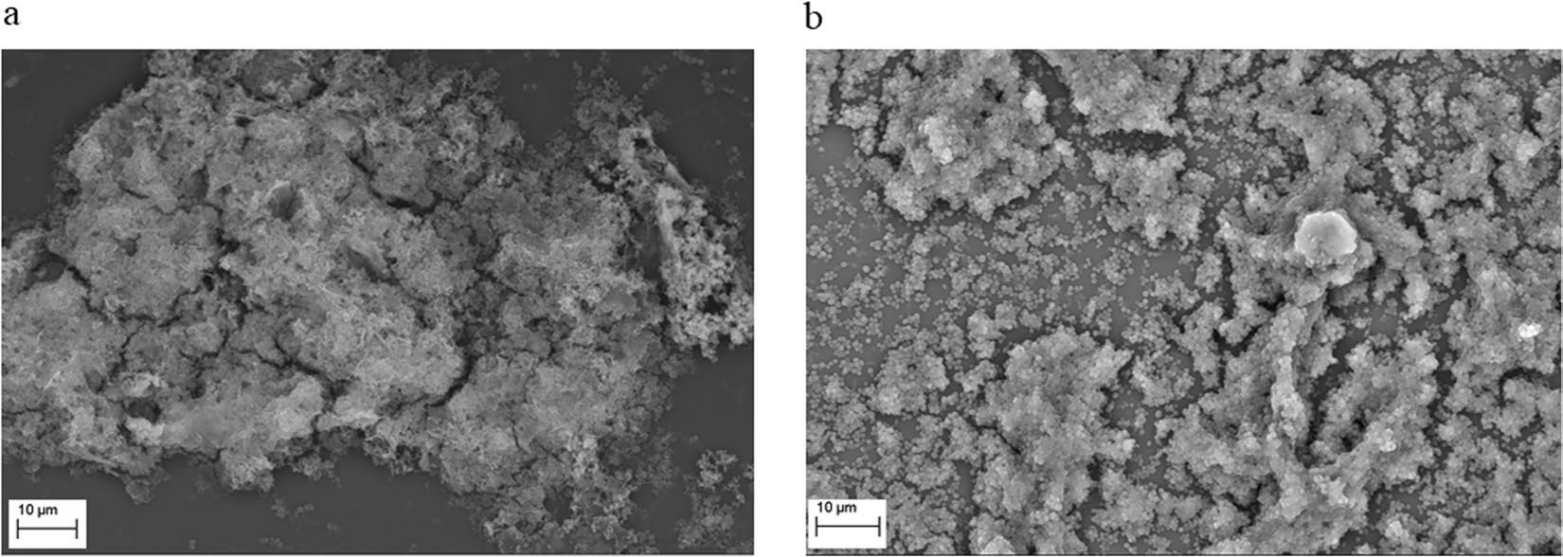
Biofilm formed by (**a**) *P. aeruginosa* and (**b**) *S. aureus* (mag. 2,500x and 2,340x, respectively).

Both investigated bacterial species were able to form biofilm structures in the experimental settings applied as proven by means of SEM. As observed in Fig. 1, *P. aeruginosa* and *S. aureus* formed multi-cellular and multi-layer biofilm, partially covered with extra-cellular matrix. Other stages of biofilm development observed included multi-layers of bacterial cells with no extracellular matrix. With the ability to biofilm formation proven, we performed a set of experiments aimed at showing the impact of RMF on antimicrobial activity and biofilm destruction.

It was also confirmed, that applied RMF had no significant impact on viability of L929 fibroblast cell line (Supplementary Fig. S1). In comparison to RMF-unexposed control samples, average percentile reduction of viability was 7.6 ± 2.4 when the highest frequency of RMF and the longest time of exposure (i.e. RMF of 50 Hz for 1 h) was used. All other observed differences in L929 fibroblast viability between unexposed control and RMF-exposed samples were also of minor nature and statistically insignificant (p < 0.05, Tukey’s HSD test).

### Experimental setting I, antibiotics: exposure of biofilm with gentamicin or ciprofloxacin-supplemented medium to RMF of 10–50 Hz for 1 h

**Figure 2:**
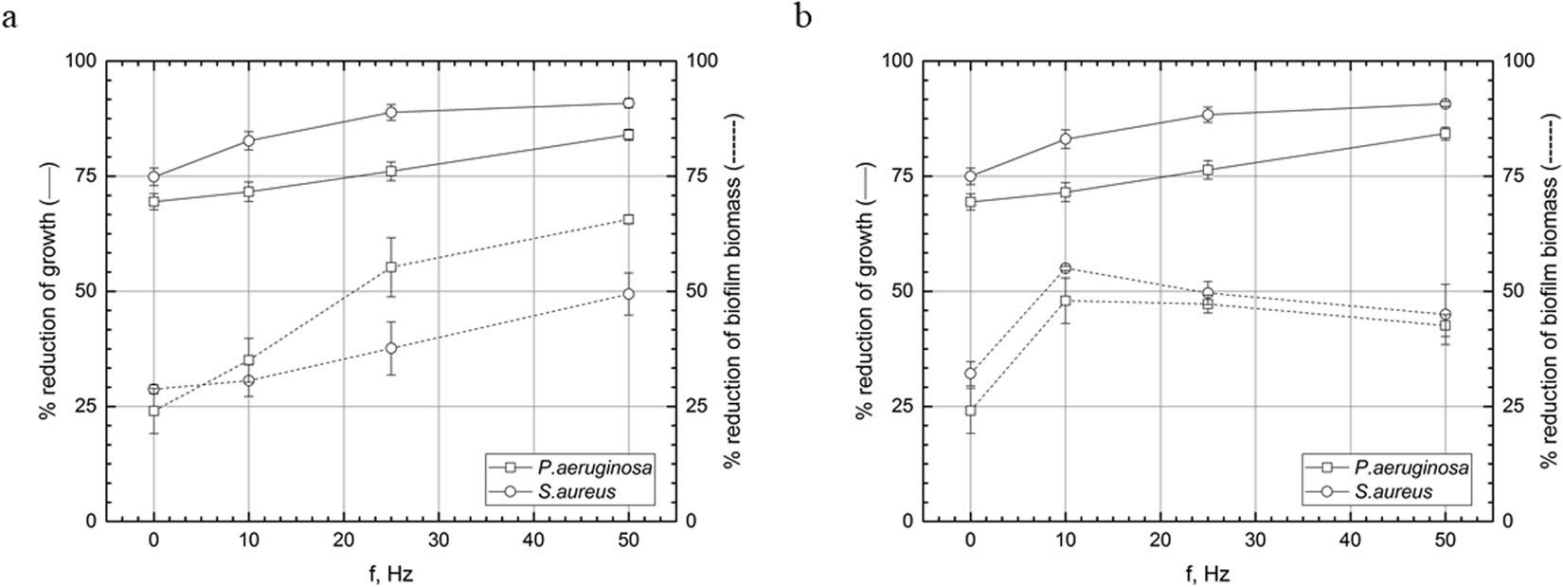
Reduction [%] of growth and biofilm biomass of microorganisms in cultures with (**a**) gentamicin and (**b**) ciprofloxacin after 1 h exposure to RMF depending on RMF frequencies. The results are presented as % reduction of growth and biofilm biomass in cultures with antimicrobial in comparison to the culture without antimicrobial after 1 h exposure to RMF and expressed as a mean ± SEM calculated from the four repetitions of the experiment.

Coupled activity of RMF and gentamicin led to a reduction of biofilm biomass (cells + extracellular layers) and count of living pseudomonal and staphylococcal cells in comparison to samples treated with gentamicin but non-subjected to RMF (0 Hz) (Fig. 2a,b, Supplementary Fig. S2a,b). In case of pseudomonal biofilm biomass, inhibiting effect was statistically significant for 10, 25, 50 Hz frequencies applied (p < 0.01, p < 0.001, p < 0.001, respectively, Tukey’s HSD test). Also differences between particular frequencies were statistically significant (p < 0.05, Tukey’s HSD test). Application of 10 and 25 Hz frequency was inefficient in case of *S. aureus* biofilm biomass, while application of 50 Hz led to significantly increased gentamycin activity (p < 0.001, Tukey’s HSD test). In case of pseudomonal cell count, statistically significant (p < 0.001, Tukey’s HSD test) inhibition of growth occurred when frequencies higher than 10 Hz were applied, while RMF acted stronger on *S. aureus* – all applied RMF frequencies led to a statistically significant (p < 0.001, Tukey’s HSD test) reduction in the staphylococcal cell count.

Similar results regarding pseudomonal cell count were obtained when ciprofloxacin was used. RMF of 10 Hz did not contribute to a statistically significant reduction of pseudomonal cell count, while higher frequencies did contribute to this (p < 0.05, Tukey’s HSD test). The pseudomonal and staphylococcal biomass as well as staphylococcal cell count were reduced significantly (p < 0.001, Tukey’s HSD test) in all RMF frequencies applied. The results of statistical analyses are shown in Table Supplementary S1 and S2.

### Experimental setting II, antibiotics (biofilm subjected to RMF of 10–50 Hz for 1 h then incubated for 17 h in medium supplemented with antibiotic)

**Figure 3:**
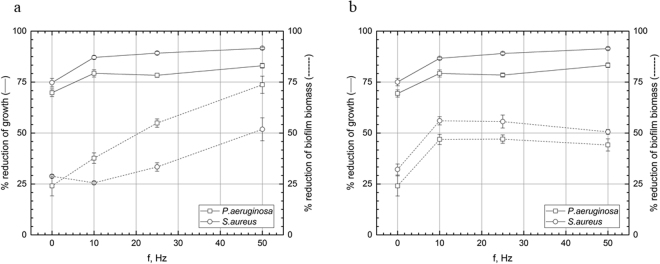
Reduction [%] of growth and biofilm biomass of microorganisms in cultures subjected to the RMF for 1 h then incubated for 17 h in medium supplemented with (**a**) gentamicin and (**b**) ciprofloxacin. The results are presented as % reduction of growth and biofilm biomass in cultures with antimicrobial in comparison to the culture without antimicrobial after 1 h exposure to RMF and expressed as a mean ± SEM calculated from the four repetitions of the experiment.

RMF of all applied frequencies elevated considerable gentamicin efficacy against *P. aeruginosa* (Fig. 3a, Supplementary Fig. S3a). RMF acted stronger on a biofilm biomass than on cells of this pathogen, e.g. substantial differences in growth inhibition were observed between all frequencies applied. In case of *S. aureus* biomass, only 50 Hz RMF frequency did increase significantly gentamicin activity (p < 0.001, Tukey’s HSD test). As for ciprofloxacin, applied spectrum of frequencies acted efficiently against pseudomonal cells and biomass (Fig. 3b, Supplementary Fig. S3b). Also, all frequencies applied increased ciprofloxacin activity significantly (p < 0.001, Tukey’s HSD test) against staphylococcal biomass and staphylococcal cell count. The results of statistical analyses are shown in Table Supplementary S1 and S2.

### Experimental setting I, antiseptics: exposition of biofilm with antiseptic to RMF of 10–50 Hz for 5 min

**Figure 4:**
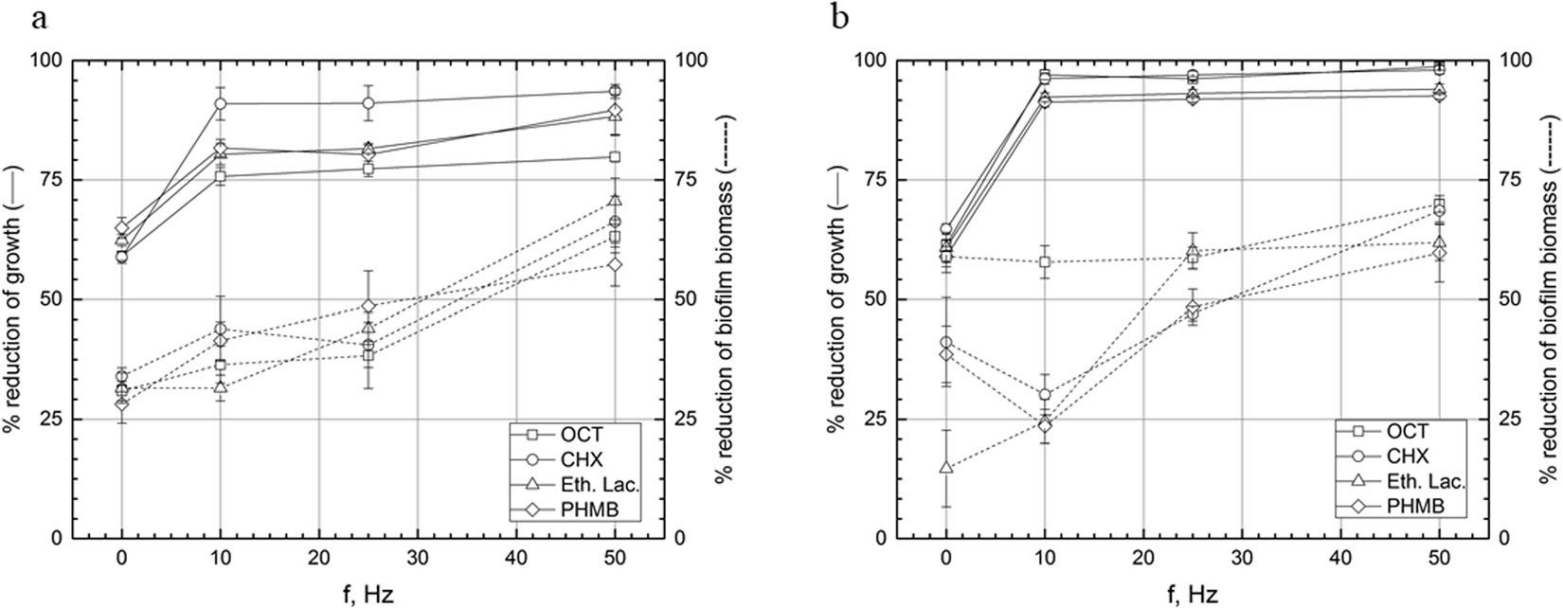
Reduction [%] of growth and biofilm biomass of (**a**) *P. aeruginosa* and (**b**) *S. aureus* in cultures supplemented with antiseptics after 5 min exposure to RMF. The results are presented as % reduction of growth and biofilm biomass in cultures with antimicrobial in comparison to the culture without antimicrobial after 5 min exposure to RMF and expressed as a mean ± SEM calculated from the four repetitions of the experiment; OCT – octenisept; CHX – chlorhexidine; Eth.Lac. – ethacridine lactate; PHMB – polyhexamethylene biguanide.

The observed trend of biofilm reduction was different for antiseptics in comparison to antibiotics. The most potent antiseptic, able to reduce pseudomonal biofilm biomass in all RMF frequencies applied was PHMB (significantly increased activity in 10, 25 and 50 Hz, p < 0.001, Tukey’s HSD test), then ethacridine lactate (significantly increased activity in 25 and 50 Hz, p < 0.001, Tukey’s HSD test) and CHX (substantial biomass reduction in 10 and 50 Hz frequency, p < 0.001, Tukey’s HSD test). In case of OCT, application of only 50 Hz frequency contributed to increased activity (p < 0.001, Tukey’s HSD test) (Fig. 4a, Supplementary Fig. S4a). However, all antiseptics reduced significantly (p < 0.001, Tukey’s HSD test) the count of pseudomonal cells regardless RMF frequency applied. The same trend, concerning cell count was observed for *S. aureus* (Fig. 4b, Supplementary Fig. S4b). With regard to staphylococcal biofilm biomass, PHMB and ethacridine lactate worked more efficiently in all RMF frequencies applied, while 50 Hz frequency was needed to significantly increase OCT activity (p < 0.001, Tukey’s HSD test). CHX worked significantly more efficiently in the presence of RMF of 10 and 50 Hz (p < 0.001, Tukey’s HSD test). The results of statistical analyses are shown in Table Supplementary S1 and S2.

### Experimental setting II, antiseptics: biofilm subjected to RMF of 10–50 Hz for 1 h then incubated for 5 min in antiseptic

**Figure 5:**
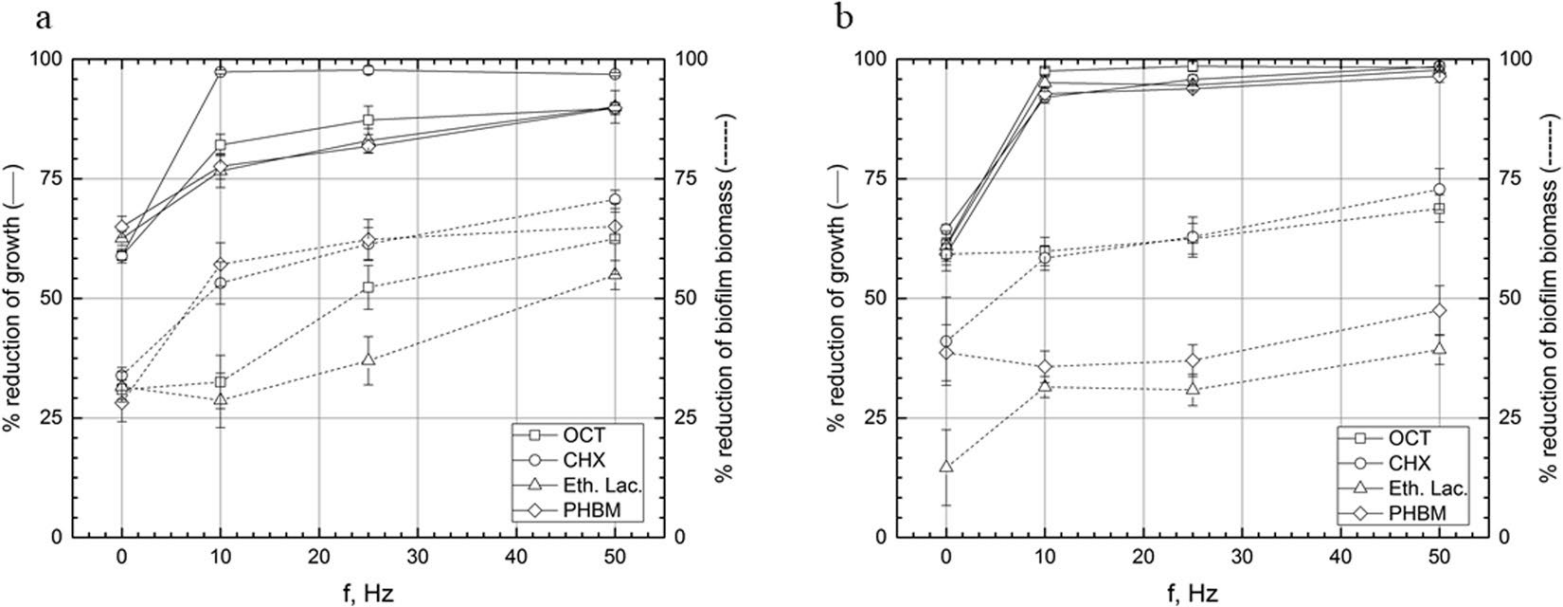
Reduction [%] of growth and biofilm biomass of (**a**) *P*. *aeruginosa* and (**b**) *S. aureus* in cultures subjected to the RMF for 1 h then incubated for 5 min in medium supplemented with antiseptics. The results are presented as % reduction of growth and biofilm biomass in cultures with antimicrobial in comparison to the culture without antimicrobial after 5 min exposure to RMF and expressed as a mean ± SEM calculated from the four repetitions of the experiment; OCT – octenisept; CHX – chlorhexidine; Eth.Lac. – ethacridine lactate; PHMB – polyhexamethylene biguanide.

Interestingly, the same trend was observed for staphylococcal and pseudomonal cell count, when they were first “sensitized” by RMF and then subjected to antiseptic activity (Fig. 5a,b, Supplementary Fig. S5a,b). Regardless from antiseptic and RMF frequency, all reductions in above-mentioned parameters were statistically significant (p < 0.001, Tukey’s HSD test). And again, differences in efficacy of antiseptics/RMF were seen, when biofilm biomass was parameter analyzed. When *P. aeruginosa* was tested, PHMB and CHX worked effectively with all frequencies of RMF applied (p < 0.001, Tukey’s HSD test); at 10 and 25 Hz, only ethacridine lactate efficacy was not elevated significantly. Increase of RMF frequency to 50 Hz allowed this antiseptic to eradicate pseudomonal biomass in a substantially higher manner in comparison to RMF-unexposed samples (p < 0.001, Tukey’s HSD test).

When *S. aureus* biomass was sensitized by RMF of 10 Hz and 25 Hz frequency, only chlorhexidine and ethacridine lactate were significantly (p < 0.001, Tukey’s HSD test) efficient; application of 50 Hz led to substantial biomass reduction in the case of all tested antiseptics. The results of statistical analyses are shown in Table Supplementary S1 and S2.

## Discussion

The results presented herein demonstrate statistically significant increases of antimicrobial-mediated reduction of biofilm exposed or sensitized by RMF. RMF effects appear to be related to multiple factors present in our experimental setting, namely: microbial species, cellular morphology, type of extracellular matrices produced, RMF frequency and mode of application, type of antimicrobial used and the medium. The rationale justifying this statement is the fact that magnetic fields influence all electrically charged particles^24^.

In the present work we observed some differences with regard to results when experiments were carried out by means of various tests, namely MTT (3-(4,5-Dimethylthiazol-2-yl)-2,5-diphenyltetrazolium bromide and CV (Crystal Violet) assay. In our opinion, these differences were related to the different biofilm aspects measured by the tests. Biofilm consists of bacterial cells and extracellular matrix. MTT measures cell count (indirectly by assessment of their metabolic activity), while CV assay provides data on both cell number and matrix amount (in other words, CV measures the amount of whole biofilm biomass).

Microbial biofilm adherence is mediated by adhesins, extracellular matrix, and by cells. All these factors possess electrical charge – for example pseudomonal matrix (that consists of alginate) as well as pseudomonal external LPS structures and adhesins are strongly negatively charged^25,26^. Also, staphylococcal biofilm and cell components are not electrically indifferent^27^. Interestingly, we did not observe significant differences in antimicrobial activity of tested substances with regard to the two bacterial species investigated, e.g. *P. aeruginosa* and *S. aureus*. These two species differ with regard to their cellular shape (Pseudomonas are rods, while Staphylococci are round clusters) and biofilm matrix produced. In our earlier work^20^ we showed that RMF inhibits various cellular activities of Gram (−) pathogens (*A. baumanii*, *P. aeruginosa*) stronger than Gram (+) *S. aureus*. However, we cannot make direct comparisons between our former and the present experimental setting, because of differences related with time of exposure to RMF. Therefore, we assume that lack of differences observed this time might be related to the antimicrobials used and their mode of action, however further experiments using more bacterial species of various sizes and shapes as well as various antimicrobials are required to fully elucidate the trends observed. Other teams that worked on a different type of electromagnetic field (e.g. static, pulsed) but on the same pathogens (*S. aureus* and *P. aeruginosa*) observed that application of electromagnetic fields (even as a potential treatment measure alone) leads to clear decreases in bacterial counts. They speculated that it might be related to formation of free radicals in the liquid environment that are detrimental for bacteria^28^ or/and related to electrophoresis, ionophoresis and electroporesis, that overcome the biofilm biomass and cell wall barriers^29^.

Microbial transport of nutrients and metabolites relies on ion exchange canals; ions are also present inside of bacterial cytoplasm and in the external environment. Bearing in mind that all biological structures are somewhat electrically charged we can, at least partially understand discrepancies between results of other teams investigating magnetic fields. The complexity and number of microbial structures and factors that might be influenced by a magnetic field is tremendous and it may – with consideration to their reciprocal interactions – contribute to the whole spectrum of results observed herein. Also, because opposite poles rotate around a certain point, the charged molecules present in a medium will move in an unpredictable, Brownian-type motion. Indeed, one of the RMF application results is high mixing of medium, increased oxygenation and temperature rise (in our experimental setting temperature was kept at constant level of 37 °C by external coolers) due to increased particle movement^30,31^

Other teams have studied the impact of other type of electromagnetic fields (static, pulsed, etc.) on biofilms, and observed alterations of these structures as a result. They explain findings by pH modifications, better transportation of antimicrobial agents into the biofilm, production of biocide ions, and hyperoxygenation. This last parameter is believed to increase the activity of certain antibiotics against bacteria^32^.

In our work, we observed elevated activity of various antimicrobials against staphylococcal and pseudomonal biofilms when they were exposed to RMF of 10–50 Hz frequency. The higher the frequency, the greater the antimicrobial effect observed. This phenomenon might be due to a direct correlation between magnetic induction and mixing within experimental settings. In other words, the more active the magnetic field was, the more particles of antimicrobial reached bacterial cells within biofilm layers. Also, higher frequency might lead to electroporation and damage of bacterial membranes, enabling antimicrobials to reach the cytoplasm. Mechanisms of RMF action are not fully elucidated and we are presently unable to provide specific explanations for the observed correlation between frequency and antimicrobial effect without further studies, which we will address in the future.

Also, if we follow the old dogma that biofilms owe their high antimicrobial tolerance to the extracellular matrix, where antimicrobials are retained and cannot reach the cells hidden underneath^33^, the question to be addressed is – what type of changes in biofilm matrix are induced by a RMF? We think that a previous study by our team may provide an answer to this question. We observed that cellulose-based biofilm matrix of *Komagateibacter xylinus* changes its properties when incubated in an RMF generator. *K*. *xylinus* bio-cellulose matrix is a great experimental model for studying biofilm matrices, because contrary to a majority of clinically relevant bacteria, it forms biofilm structures of circa 2.5 cm thickness and a size limited to the surface of the container where it is cultured. Thus, it allows the investigation of various matrix parameters with relatively simple, macroscopic methods. Although the size of *K. xylinus* biofilm matrix is macroscopic, its building blocks of cellulose fibers are a few micrometers in diameter. One property of bacterial cellulose is its ability to retain water and other fluids within its structure. It is estimated that bio-cellulose can absorb a volume of water exceeding up to 100x its dry mass^34^. In our publication from 2016 we showed that bio-cellulose forming under RMF exposure can absorb 33% more water than RMF-unexposed cellulose. Moreover, using SEM we observed that fibrils forming RMF-exposed cellulose matrix differs from RMF-unexposed cellulose fibrils – and is dependent on the RMF frequency and exposure time as some matrices consisted of thicker and denser fibrils, while some were thinner than fibrils of control samples. Thus, it can be stated that RMF had a significant impact on cellulose fibril properties and their cross-linkage.

Extrapolation of our results to matrices of other biofilms may help in the understanding of the increased penetrability of antimicrobials as we observed. Many clinically significant bacteria possess cellulose content in their extracellular matrix (*Enterobacter* spp., *Pseudomonas* spp., *Burkholderia* spp., *Escherichia* spp. are the most prominent examples), and all biofilm matrices resemble nets of various pore size^35^. Thus, there are at least a few possible explanations of better antimicrobial penetrability in a RMF-exposed environment. First is an altered (diminished) matrix fibril cross-linkage, resulting in higher pore size and better antimicrobial penetration. Second is that matrix fibrils display an altered electrical charge due to RMF activity and thus they display lower affinity to antimicrobials. Third is a combination of the two above, and fourth focuses on the mixing effect of RMF that also allows antimicrobials to reach deeper layers of biofilm in a more efficient manner. Another possibility is the least likely of the aforementioned, that we purposely used antimicrobials of various (cationic or anionic) characters to test. We did not however observe any meaningful differences between increased activity regarding cationic or anionic character of tested antimicrobials. If RMF application altered an electrical charge of matrix components, we would observe higher activity of one type of antimicrobial over the other regarding their respective charge.

**Figure 6:**
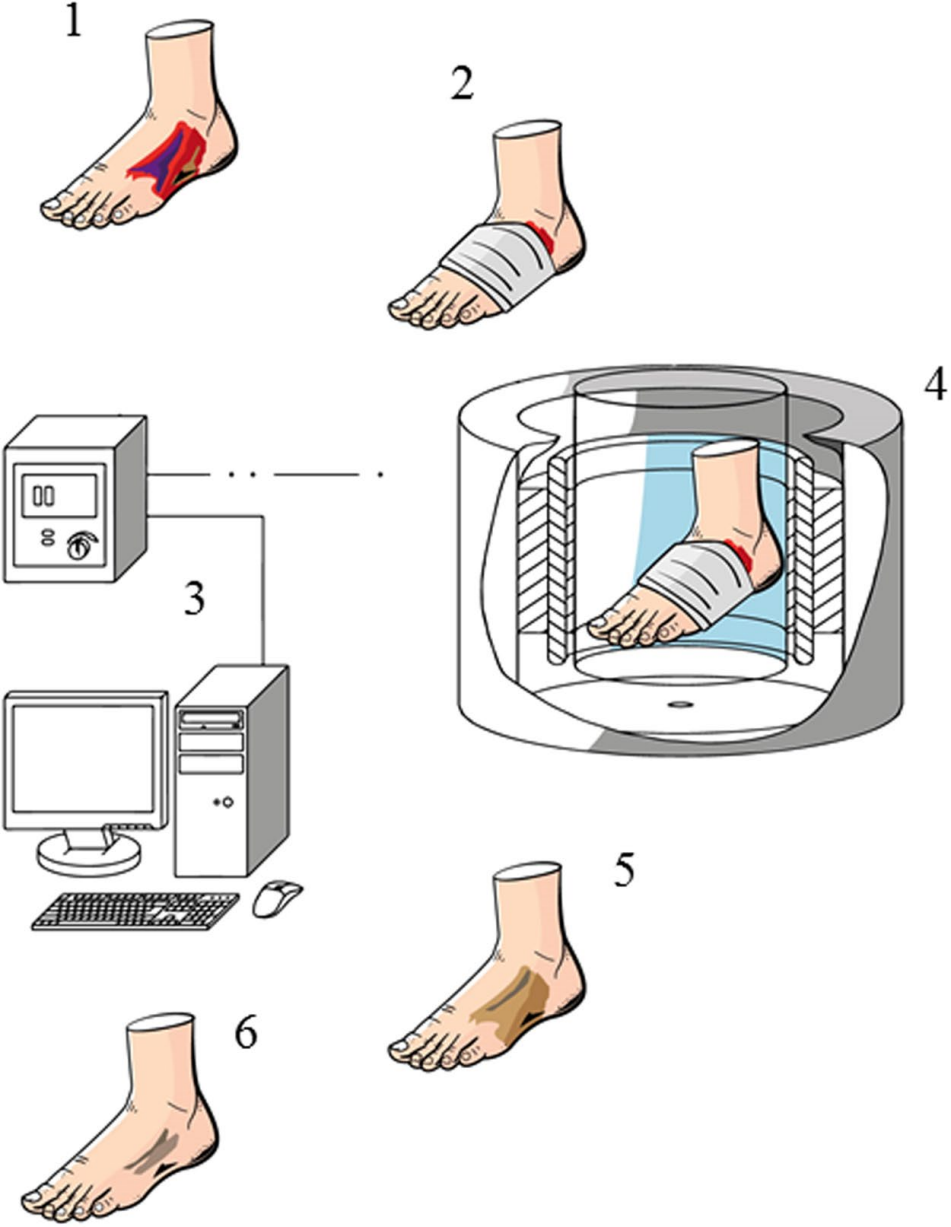
Visualization of possible treatment procedure using RMF to treat chronic wounds. 1 – diabetic foot ulcer; 2 – diabetic foot ulcer covered with a dressing saturated with antiseptic; 3 – personal computer and transistor inverter; 4 – treatment of a diabetic foot ulcer in a RMF generator; 5 – eradication of infection; 6 – enhanced recovery.

One should bear in mind that even Nikolai Tesla, one of the most accomplished electromagnetic field researchers, said once that the nature of magnetic fields is one of the most fundamental, if not a fundamental, riddles of nature^36^. We should thus recognize that for now and likely for a long time, science will continue to use magnetic field properties without understanding their nature. However, independent from an actual causative factor for results obtained herein, our findings are of high translational value because of ease and relevance of potential clinical applications. We observed circa 20–50% increase in the antibiofilm efficacy of various antiseptics expressed as an ability to diminish biofilm biomass (cells + matrix) and reducing biofilm forming cell count. Similar results were obtained for two antibiotics tested, namely gentamicin and ciprofloxacin. These results are of tremendous potential meaning with regard not only for hospital finances but also for the rationale for antibiotic therapy. Application of RMF coupled with antimicrobials in the treatment of chronic wounds clinically may reduce costs related with antiseptic and antibiotic therapy and correlate with a lower usage of these antimicrobials, reducing risk of resistance by bacteria and toxic side-effects. A possible treatment procedure using RMF to treat chronic wounds is presented in Fig. 6.

Chronic wounds with protracted infection are a serious clinical problem. For example, 25% of patients with diabetes will develop a diabetic foot ulcer at some point and in the USA alone there are 23 million people with diabetes^37^. Some of these ulcers will progress to diabetic foot osteomyelitis, which is associated with limb-threatening and life-threatening sequelae and caused by *S. aureus*, which is why we tested this key pathogen herein. Chronic leg ulceration affects circa 1% of the adult population^38^. Other chronic wound types include bed sores and burn wounds. Thus, the total number of patients suffering from chronic wounds at risk of infection may include hundreds of millions of people worldwide. The costs of treatment are difficult to calculate and they depend on many factors, including type of wound, patient’s general status, antibiotic and antiseptic therapy course, involvement of hospital staff in treatment, care and rehabilitation procedures to name a few. A roughly estimated financial burden of infection treatment may reach several thousand dollars per patient^39^. An estimated cost of our prototypical RMF generator intended to be used for hospital or ambulatory chronic wound treatment is about 5000 US dollars. Thus, the use of such a device could contribute to a significant savings and improved morbidity and mortality.

There are many issues that need to be resolved before such an approach as presented herein could be applied in a hospital setting for the treatment of chronic wounds, and experiments in animal models are a necessary next-step to exclude a risk for adverse events and to demonstrate safety and efficacy. To the best of our knowledge, there are no experiments on RMF impact on animals performed to date, however investigations showing a beneficial impact of magnetic fields on eukaryotic cell cultures *in vitro* and on rat behavior have been performed^16,40^. Therefore, well-controlled animal studies are needed for *in vivo* testing of RMF generators as applied to chronic wound treatment. Preliminary results of RMF impact on L929 fibroblast viability (Supplementary Fig. S1) show lack of detrimental effect of this type of field on wound healing cells. However, one should remember that prolonged exposure of pathogens to RMF may lead to faster growth in particular cases^20^. Further testing and development of this technology has the potential to reduce the morbidity and mortality associated with chronic infected wounds, particularly for conditions such as diabetic foot ulcers and leg ulcers which can lead to loss of limb or life.

## Materials and Methods

### Biofilm formation

For experimental purposes, *S. aureus* ATCC6538 and *P. aeruginosa* ATCC15442 were used. Initially, bacteria were plated onto Columbia Agar with 5% sheep blood and cultivated for 24 h at 37 °C. After incubation, one colony forming unit (CFU) of each bacterial species was transferred into 10 mL of Tryptic Soy Broth and incubated another 24 h at 37 °C with shaking (200 rpm). Next, cultures were diluted in TSB broth to obtain the same optical density (OD) equals 1 × 10^8^ CFU/mL. In the next step, 10 mL of the resulting bacterial suspension was transferred to inoculate 100 mL of TSB supplemented with 1% glucose, mixed and added to 24-well plate (1 µL into each well). To obtain biofilm, the plates with bacterial suspension were incubated for 48 h at 37 °C with replacement of the medium every 12 h.

### Antimicrobials

Prior to the addition of the substances with antimicrobial activity, the TSB medium was removed and the wells with biofilm were washed two times with PBS buffer. Then, 1 mL of PBS containing two antibiotics: gentamicin and ciprofloxacin (Sigma-Aldrich, Germany) and four antiseptics: octenisept (Schulke Mayer, Germany), chlorhexidine (Amara, Poland), ethacridine lactate (ProLab, Poland), polyhexamethylene biguanide (B Braun, Germany) were added to each well of 24-well plate. As it was determined in the initial step of the study, the concentration of antimicrobials used in the experiment caused the reduction of growth of bacteria in biofilm and biofilm biomass of approximately 50–75% and 18–59% respectively (Table 1). The final concentration of antimicrobial substances were as follows: (a) gentamicin for *S. aureus* – 30 mg/L, for *P. aeruginosa* −1.3 mg/L (solution in PBS, v/w); (b) ciprofloxacin for *S. aureus* – 1.5 mg/mL, for *P. aeruginosa* – 0.024 mg/mL (solution in PBS, initially dissolved in DMSO, v/w); (c) octenisept for *S. aureus* – 0.0975%, for *P. aeruginosa* – 1.56% (solution in PBS, v/v); (d) chlorhexidine for *S. aureus* – 0.039%, for *P. aeruginosa* – 0.039% (solution in PBS, v/v); (e) ethacridine lactate for *S. aureus* – 0.03%, for *P. aeruginosa* – 0.005% (solution in PBS, v/v); (f) polyhexamethylene biguanide for *S. aureus* – 0.02%, for *P. aeruginosa* – 0.005% (solution in PBS, v/v).

**Table 1:** Reduction of growth of bacteria in biofilm and biofilm biomass at the concentration of antimicrobials used in the experiment.

Antimicrobial	*S. aureus*			*P. aeruginosa*		
	Concentration	%RG	%RB	Concentration	%RG	%RB
Gen	30.000 mg/L	75	29	1.300 mg/L	69	24
Cip	1.500 mg/mL	62	32	0.024 mg/mL	50	24
OCT	0.0975%	61	59	1.5600%	59	31
CHX	0.0390%	65	41	0.0390%	59	34
Eth.Lac.	0.0300%	61	18	0.0050%	63	32
PHMB	0.0200%	59	39	0.0050%	65	28

%RG – % reduction of growth of bacteria in biofilm; %RB – % reduction of biofilm biomass. Gen – gentamicin; Cip – ciprofloxacin; OCT – octenisept; CHX – chlorhexidine; Eth.Lac.– ethacridine lactate; PHMB – polyhexamethylene biguanide.

### Exposure to RMF

**Figure 7:**
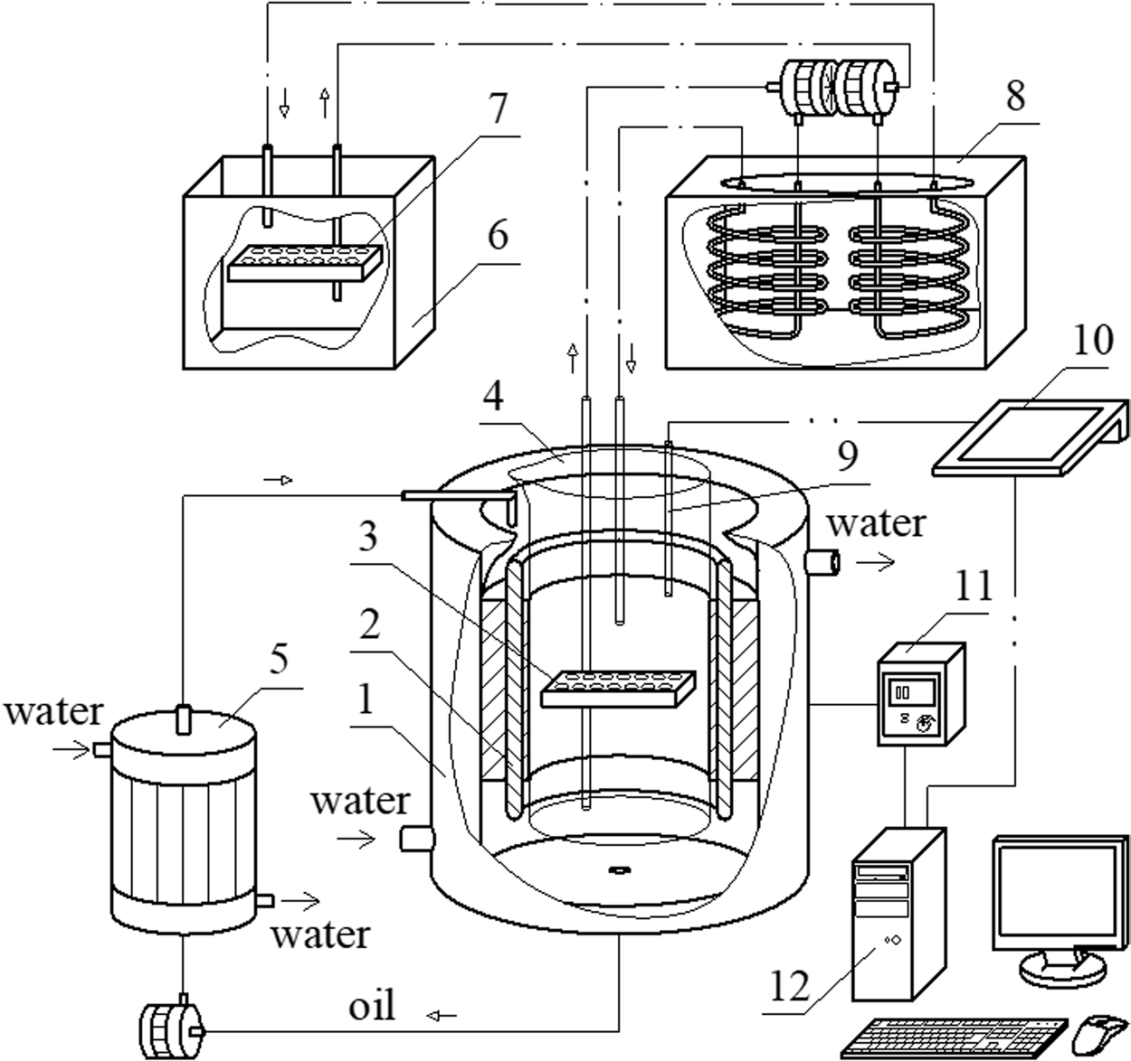
Scheme of our experimental set-up. 1 – cooling jacket, 2 – RMF generator, 3 – test plate, 4 – cylindrical glass vessel, 5 – heat exchanger for cooling system, 6 – water batch for control probes, 7 – control plate, 8 – thermostat, 9 – microprocessor temperature sensor, 10 – multifunctional meter, 11 – transistorized inverter, 12 – personal computer.

The exposure of biofilm to the RMF was carried out using a self-designed set-up, described in our previous works^17,18^ and adopted for purposes of this research. This set-up is schematically presented in Fig. 7.

The experimental set-up contained a generator for the RMF, made of a three-phase stator of an induction squirrel cage motor, and a glass container filled with demineralized water that was a water bath incubator for the tested material placed inside of it during the exposure. The glass container was axially aligned with the RMF generator and positioned symmetrically with the respect to its lower and upper ends. The RMF was generated by coils located around the cylinder, and the axes were directed along the radius. When the alternating currents are applied, the generated magnetic field rotates about the cylinder axis with the constant angular frequency of a RMF. The gaps between the electromagnetic poles and the cylindrical column were minimal.

The frequencies of the RMF (*f*) were changed by a transistorized inverter. In the experimental procedure, this frequency was changed in the range from 10 to 50 Hz. The values of magnetic induction, *B*, were detected by using a Hall probe and a personal computer. The measurement of magnetic induction at the selected RMF frequency was repeated several times and mean values of magnetic induction were calculated. Basing on the records of the magnetic induction random signals the mean values of the parameter, *B*, at each sampling point were calculated. As follows from the analysis of the calculated data, the maximal values of the magnetic induction were obtained (*f* = 10 Hz − *B*_max_ = 23 mT; *f* = 25 Hz − *B*_max_ = 29 mT; *f* = 50 Hz − *B*_max_ = 34 mT).

**Figure 8:**
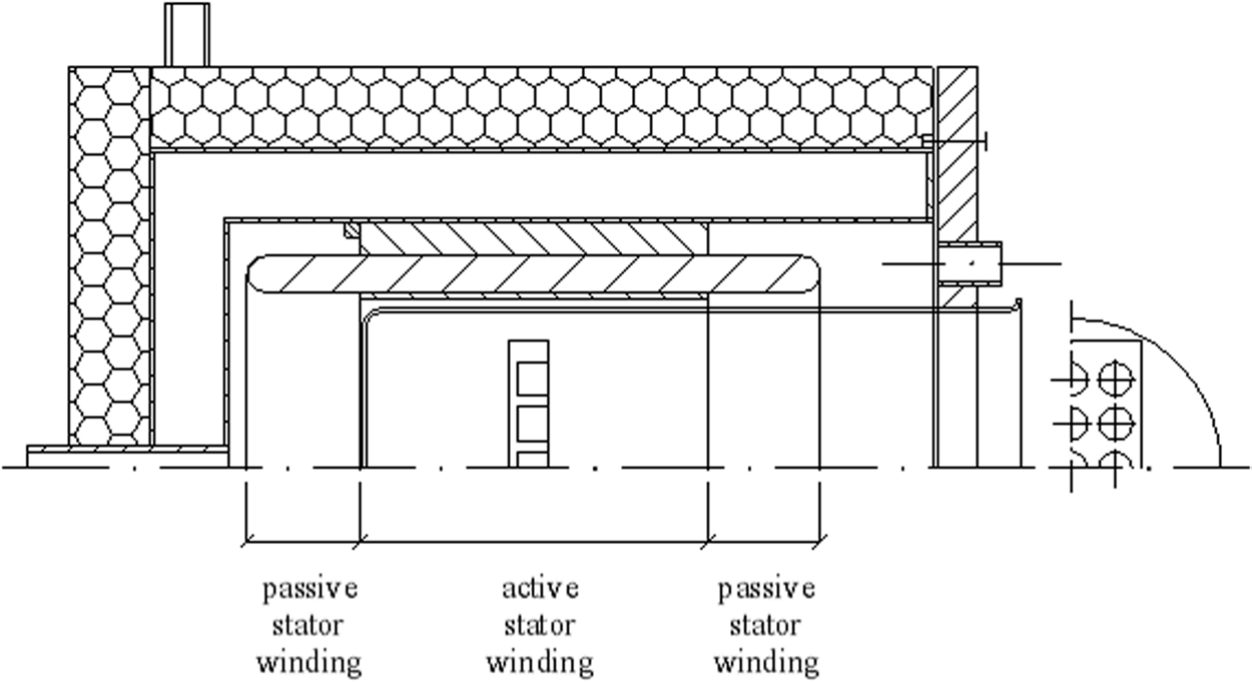
The graphical representation of the arrangement of the test plate in the cylindrical glass container.

The incubation temperature during the exposure to the RMF was controlled by a thermostat, a cooling jacket and a circulating pump. This system was used to keep the water flow rate constant in time and to set the constant temperature of the water bath (37 °C ± 0.5 °C). The temperature fluctuation inside the glass container during the experiment was measured using the microprocessor temperature sensors (LM-61B, National Semiconductor Corporation, USA). The test plates with biofilm were placed in the center of the coil of the RMF generator where the magnetic field is maximal. The graphical presentation of the arrangement of the test plate in the cylindrical glass container during the exposure to the RMF is presented in Fig. 8. The plates with biofilm formed by the same bacterial strains, incubated in the same time and under the same conditions, but without exposure to the RMF served as a control for the experiment. The controls were incubated in the water bath used to maintain the temperature of the test tubes in the RMF generator. The fluctuation of temperature was the same for tested and control samples, with deviation less than 0.5 °C. This was confirmed using a Hall probe such that the source of the RMF did not have an impact on the controls during the experiment (*B* ≤ 0.05 mT).

Two different experiments, named “Exp I” and “Exp II” were performed. The experiments were performed separately for antibiotics and antiseptics:

1. Antibiotics: Exp I) the antibiotic solution was added to the 24-well plates with biofilm, the plates were exposed to the RMF of particular frequency for 1 h, the plates were removed from the RMF generator and incubated for 17 h at 37 °C with shaking (120 rpm). Exp II) the 24-well plates with biofilm were exposed to the RMF of particular frequency for 1 h, the plates were removed from the RMF generator and antibiotic solution was added to the wells, next the plates were incubated for 17 h at 37 °C with shaking (120 rpm).
2. Antiseptics: Exp I) the antiseptic solution was added to the 24-well plates with biofilm, the plates were exposed to the RMF of particular frequency for 5 min, the plates were removed from the RMF generator, the antiseptic solution was removed and the neutralization solution (phosphate buffer 0.25 mol/L) was added. Exp II) the plates were exposed to the RMF of particular frequency for 1 h, the plates were removed from the RMF generator, the antiseptic solution was added to the 24-well plates with biofilm for 5 min, the antiseptic solution was removed and the neutralization solution was added.

### Evaluation of the reduction of growth of bacteria in biofilm (MTT assay)

Before each assay, fresh MTT solutions were prepared by dissolving 3 mg MTT (Sigma-Aldrich, Germany) in 10 mL pre-warmed (37 °C) PBS. One hundred μl PBS and 100 μL MTT solution were added to all wells. Plates were incubated in the dark for 1 h at 37 °C. In the next step, 100 µL of isopropanol was added to each well, and the plates vigorously shaken. The amount of MTT formazan formed during the incubation was measured with the Infinite 200 PRO NanoQuant reader (Tecan, Switzerland) at a wavelength of 570 nm and reference wavelength of 690 nm.

### Evaluation of the reduction of biofilm biomass (CV assay)

For fixation of the biofilms, 100 μL 99% methanol was added (15 min), after which supernatants were removed and the plates were air-dried. Then, 100 μL of a CV solution was added to all wells. After 20 min, the excess CV was removed by washing the plates under running tap water. Finally, bound CV was released by adding 150 μL of 33% acetic acid. The absorbance was measured at 590 nm using Infinite 200 PRO NanoQuant reader. All steps were carried out at room temperature.

Results of MTT, CV tests were presented as % reduction of growth and biofilm biomass of microorganisms in cultures containing antimicrobial and exposed to the RMF in comparison to the control cultures: (i) containing antimicrobial and not exposed to RMF and (ii) without antimicrobials and exposed to RMF, calculated by the formula:

$$ \% \,reduction=(\frac{(O{D}_{sample}\,-O{D}_{background})}{(O{D}_{control}-O{D}_{background})})\times 100$$

where % is a percent reduction of growth and biofilm biomass of microorganisms, OD sample is optical density obtained for biofilm from cultures containing antimicrobial and exposed to the RMF from MTT or CV assay, OD control is optical density obtained for biofilm from one of the two controls (containing antimicrobial and not exposed to RMF or without antimicrobials and exposed to RMF), OD background is optical density of the appropriate sample containing no bacteria (biofilm).

Three groups of comparison (one experimental group and two control groups) were thus applied: I – Cultures containing antimicrobial and exposed to the RMF (experimental group); II – Cultures without antimicrobials and exposed to RMF (control group I); III – Cultures containing antimicrobial and not exposed to RMF (control group II).

### Alamar blue cell viability assay of L929 fibroblasts exposed to RMF

In order to assess the possible influence of RMF on mammalian cells responsible for wound healing processes, fibroblast (L929) *in vitro* cultures exposed to RMF were evaluated using Alamar blue cell viability assay (ThermoFisher, USA). The results were compared with control samples containing the same cells, and incubated in the same conditions, but not exposed to RMF.

The fibroblast cultures used in the assay were prepared in 24 well plates with 1 mL of DMEM cell culture medium without serum (Sigma-Aldrich). The cultures were exposed to RMF of 10, 25 and 50 Hz at 37 °C in the same way as biofilm samples (for 5 minutes as samples with antiseptics and 1 h as samples with antibiotics). After exposure to RMF, 100 µL of Alamar blue was introduced to wells of the plate and cells were incubated for 3 h at 37 °C. After incubation, 200 µL of the medium with Alamar blue was transferred into wells on a black 96- well microtitre plate (Becton Dickinson and Company, USA) and the fluorescence signal was measured using microplate fluorescence reader (Synergy HTX, Biotek, USA) at wavelengths of 540 nm excitation and 590 nm emission. As a blank, pure medium was used. The results were expressed as percent of cell viability, and calculated by the formula:

$$ \% \,of\,cell\,viability=(\frac{ODs-ODb}{ODc-ODb})\times \,100$$

where OD is optical density, indexes: *s*, *b*, and *c* are referring to sample, background, and control, respectively.

### Visualization of biofilm

To additionally confirm the presence of biofilm in the wells of 24-well plates, Scanning Electron Microscopy (SEM) was performed. For this purpose, glass discs (TC Coverslip, Thermanox Inc., USA) were placed into randomly selected wells of 24-well plates during the biofilm formation step and the plates were incubated as indicated in the “Biofilm formation” section. In the next step, glass discs were removed, washed with PBS and fixed by immersion in 3% glutarate for 15 min at room temperature. The samples were rinsed twice with PBS to remove the fixative. Dehydration in increasing concentrations of ethanol (25%, 50%, 60%, 70%, 80%, 90%, and 100%) was performed for 10 min per solution. The ethanol was then rinsed off, and the samples were dried at room temperature. Next, discs were covered with gold and palladium (60:40; sputter current, 40 mA; sputter time, 50 sec) using a Quorum machine (Quorum International, Fort Worth, TX) and examined under a Zeiss EVO MA25 scanning electron microscope (SEM) (Carl Zeiss, Germany). Strains were considered able to form biofilm if they could adhere to the agar surface and if they were at least partially embedded within the extracellular biofilm matrix.

### Statistical analysis

The data obtained in this study (% reduction of growth and % reduction of biofilm biomass) were presented as mean values ± standard error of the mean (SEM) and were analyzed using two-way analysis of variance (ANOVA). The Tukey’s HSD (Honestly Significant Difference) test was used for multiple comparison of means (the post-hoc analysis) obtained from cell cultures incubated with different antimicrobials and exposed to the RMF of different frequencies. The experiment was conducted in technical triplicates and repeated four times. Differences were considered significant at a level of p < 0.05. For the effects’ determination of RMF frequencies and exposition time on fibroblast cells two-way ANOVA followed by Tukey’s HSD test for post-hoc comparisons were used. The experiments were conducted in technical triplicates and repeated four times. Differences were considered significant at a level of p < 0.05. The statistical analyses were conducted using Statistica 12.5 (StatSoft, Inc. Tulsa, OK, USA).

### Data Availability

The datasets analyzed during the current study are available from the corresponding author on reasonable request.

## Electronic supplementary material

Supplementary Information
