# Supplementary material for: Application of Rotating Magnetic Fields Increase the Activity of Antimicrobials Against Wound Biofilm Pathogens
Source: Sci Rep. 2018 Jan 9;8:167. doi: 10.1038/s41598-017-18557-7 (PMC5760636; doi:10.1038/s41598-017-18557-7)
Supplement: Supplementary file 1 — Supplementary Information [file 41598_2017_18557_MOESM1_ESM.doc]

**Supplementary information**

**Application of Rotating Magnetic Fields Increase the Activity of Antimicrobials Against Wound Biofilm Pathogens**

**Junka AF.^1^, Rakoczy R.^2^, Szymczyk P^3^., Bartoszewicz M.^1^, Sedghizadeh PP.^4^, Fijałkowski K.*^5^**

^1^ Department of Pharmaceutical Microbiology and Parasitology, Wrocław Medical University, Borowska 211A, 50-556 Wrocław, Poland

^2^Institute of Chemical Engineering and Environmental Protection Processes, Faculty of Chemical Technology and Engineering, West Pomeranian University of Technology Szczecin, Piastów 42, 71-065 Szczecin, Poland

^3^Centre for Advanced Manufacturing Technologies (CAMT/FPC), Faculty of Mechanical Engineering, Wrocław University of Science and Technology, Łukasiewicza 5,50-371 Wrocław, Poland

^4^Center for Biofilms, Ostrow School of Dentistry of University of Southern California, 925 West 34^th^, Los Angeles, California, United States of America

^5^Department of Immunology, Microbiology and Physiological Chemistry, Faculty of Biotechnology and Animal Husbandry, West Pomeranian University of Technology, Szczecin, Piastów 45, 70-311 Szczecin, Poland

Correspondence and requests for materials should be addressed to Fijałkowski K. (email: [karol.fijalkowski@zut.edu.pl](mailto:karol.fijalkowski@zut.edu.pl)).

**Supplementary information includes:**

**1. Supplementary figures**

**2. Supplementary figure legends**

**3. Results of statistical analyses**

**Fig. S1.** Viability [%] of fibroblast (L929) cell line exposed to RMF of 10, 25 and 25Hz frequency.

Viability of unexposed fibroblasts was estimated for 100% (control sample) The results are presented as a mean ± SEM calculated from the four repetitions of the experiment.

| **a** | **b** |
| --- | --- |
| 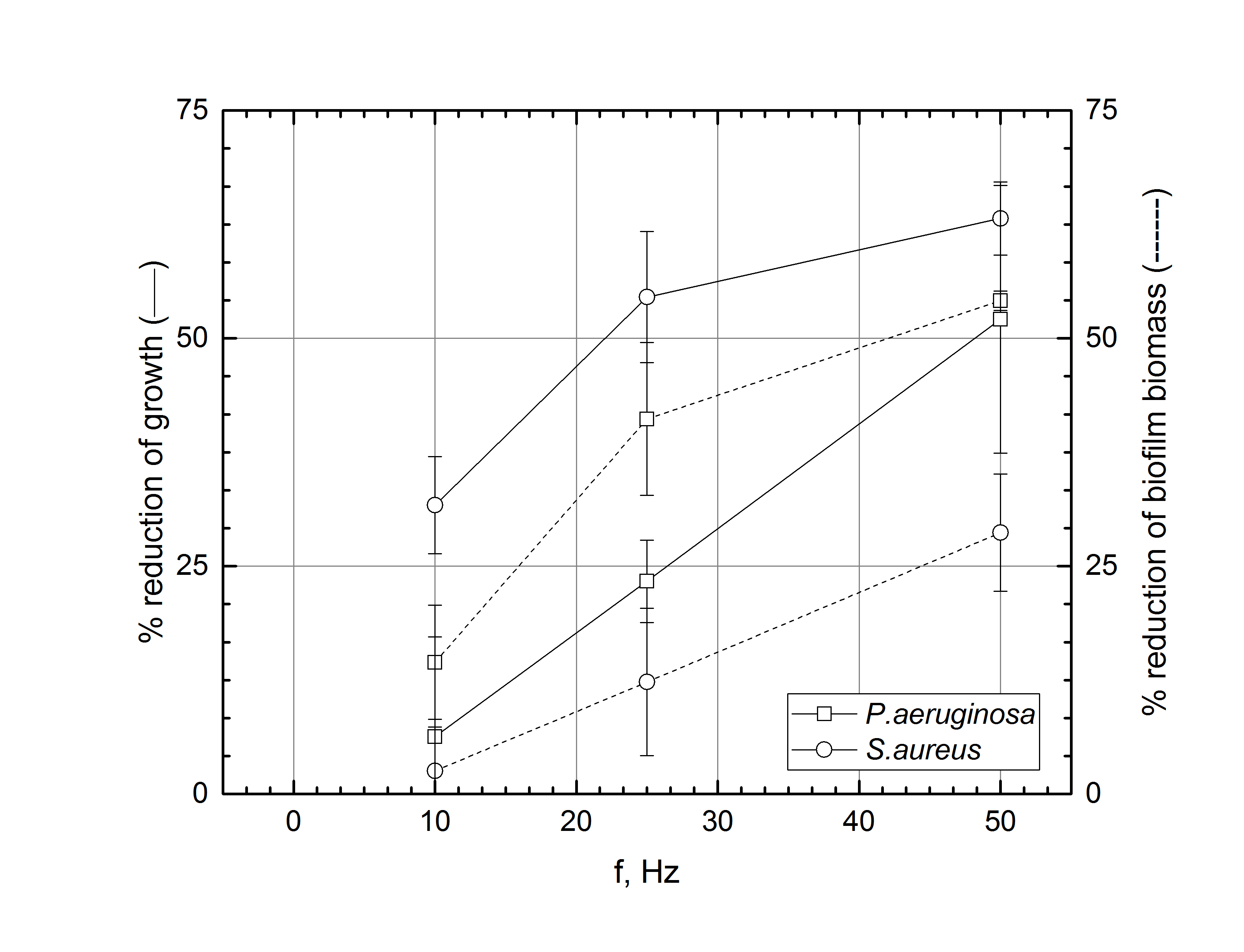 | 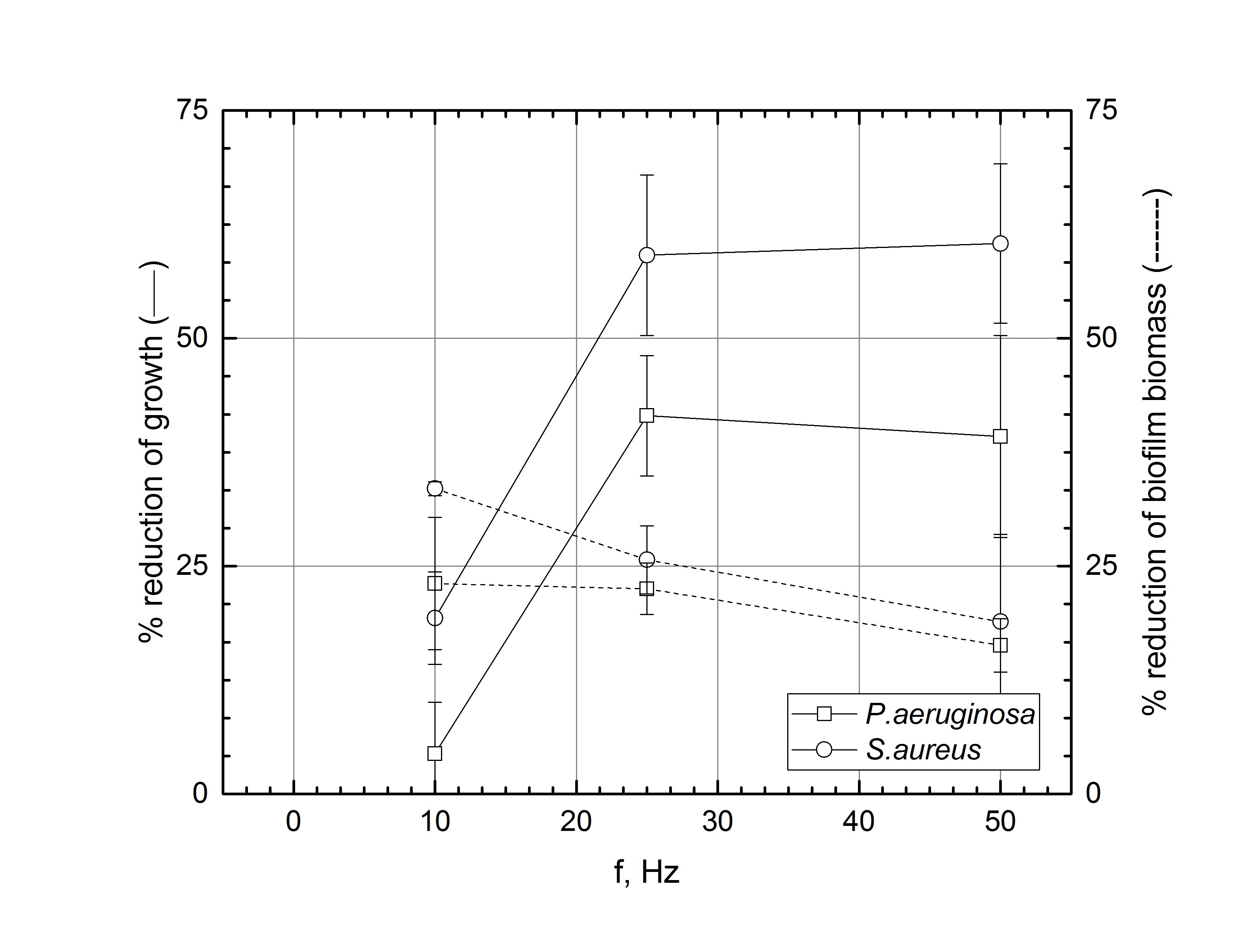 |

**Fig. S2.** Reduction [%] of growth and biofilm biomass of microorganisms in cultures with (a) gentamicin and (b) ciprofloxacin after 1 h exposure to RMF depending on RMF frequencies.

The results are presented as % reduction of growth and biofilm biomass in cultures with antimicrobial after 1 h exposure to RMF in comparison to the culture with antimicrobial not exposed to RMF and expressed as a mean ± SEM calculated from the four repetitions of the experiment.

| **a** | **b** |
| --- | --- |
| 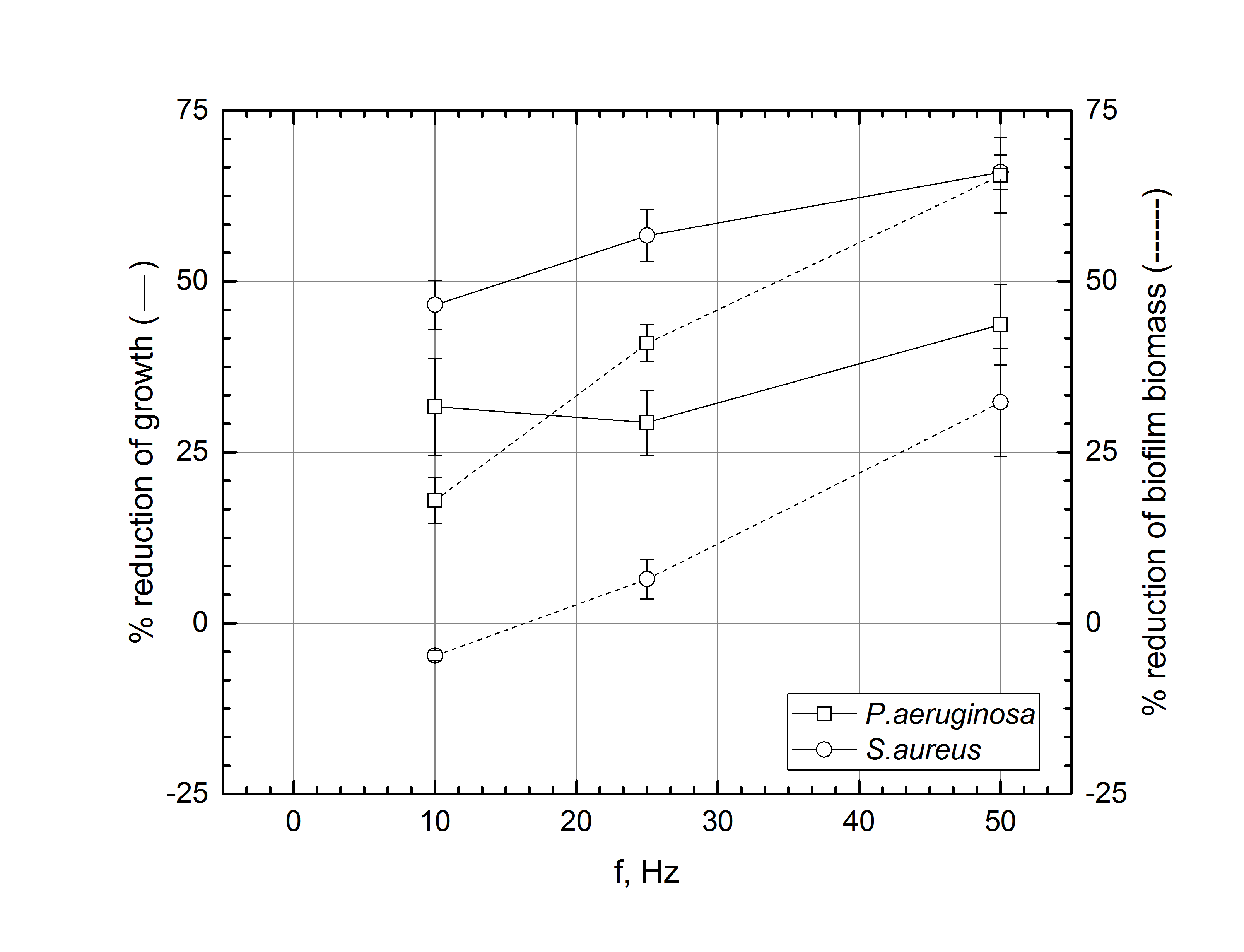 | 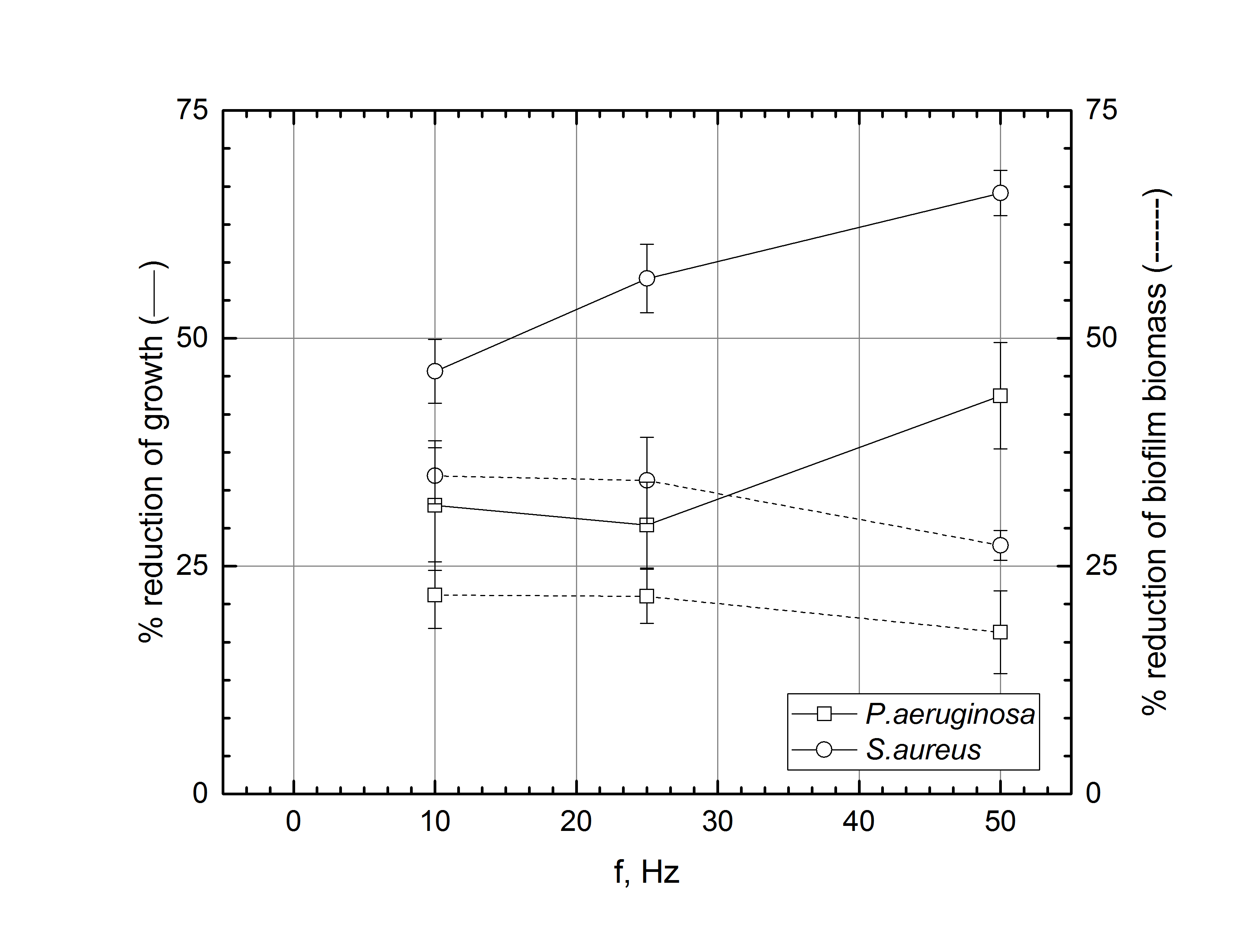 |

**Fig. S3.** Reduction [%] of growth and biofilm biomass of microorganisms in cultures subjected to RMF for 1 h then incubated for 17 h in medium supplemented with (a) gentamicin and (b) ciprofloxacin.

The results are presented as % reduction of growth and biofilm biomass in cultures subjected to RMF for 1 h then incubated for 17 h in medium supplemented with antimicrobial in comparison to the culture with antimicrobial not exposed to RMF and expressed as a mean ± SEM calculated from the four repetitions of the experiment.

| **a** | **b** |
| --- | --- |
| 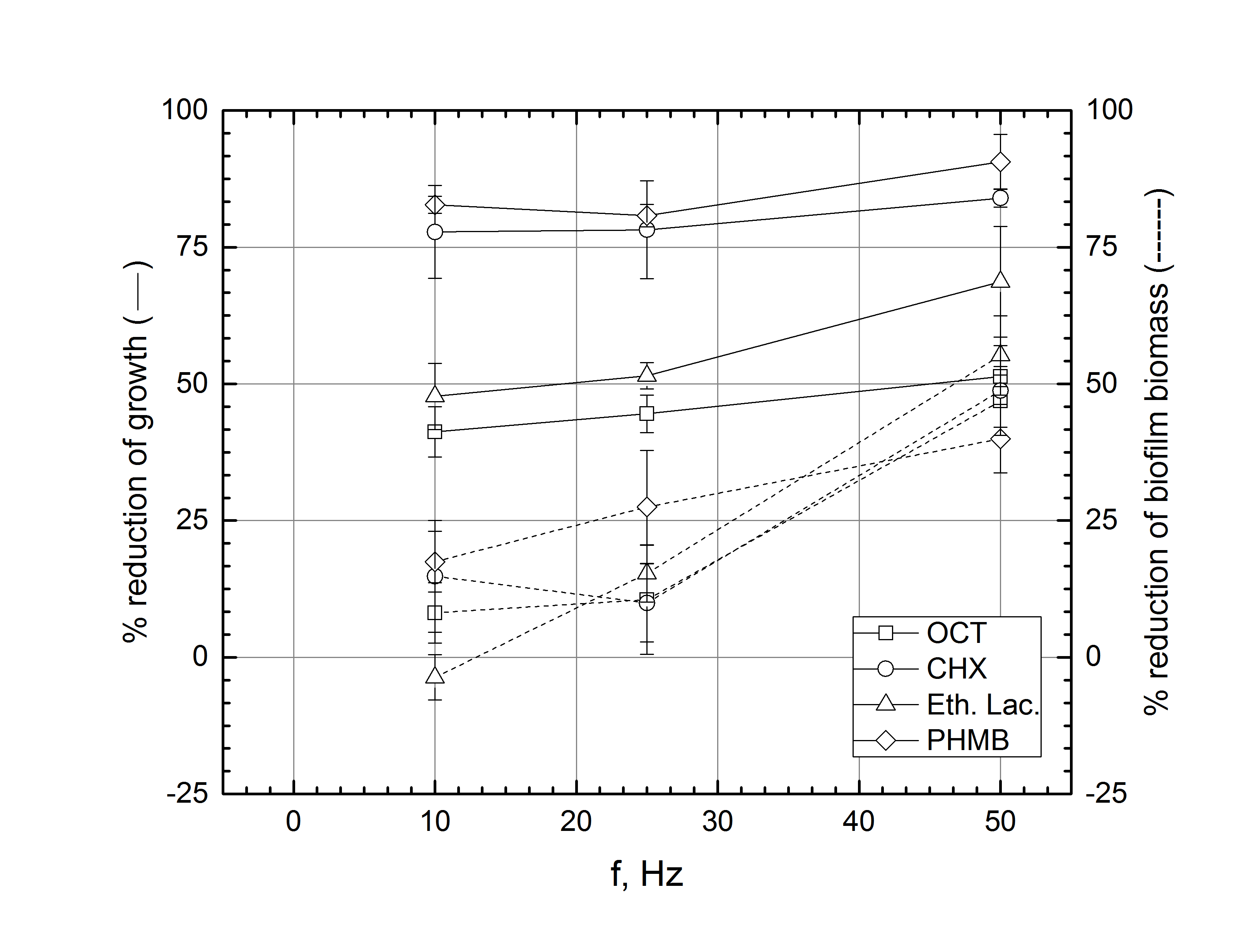 | 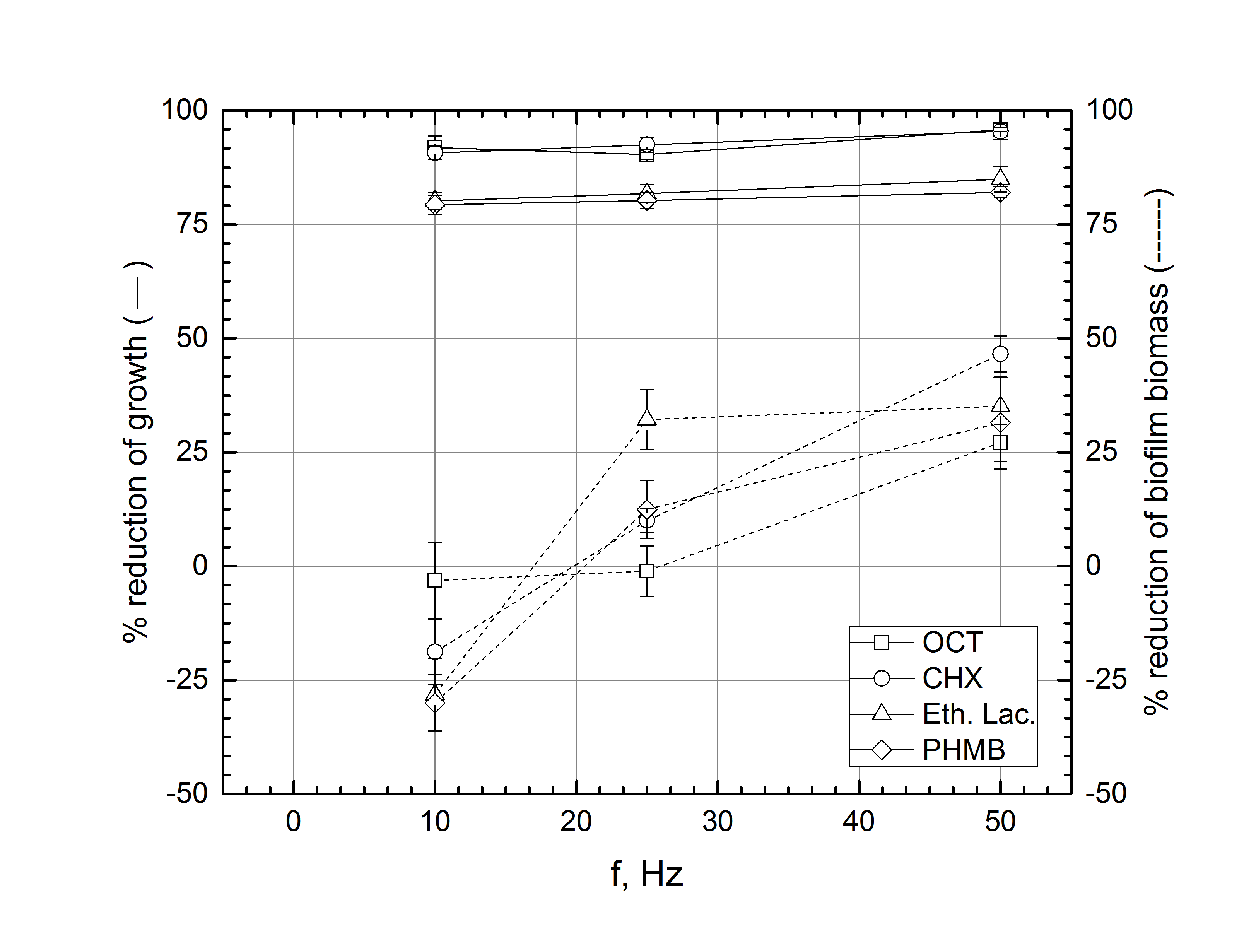 |

**Fig. S4.** Reduction [%] of growth and biofilm biomass of (a) *P. aeruginosa* and (b) *S. aureus* in cultures supplemented with antiseptics after 5 min exposure to RMF.

The results are presented as % reduction of growth and biofilm biomass in cultures supplemented with antimicrobial after 5 min exposure to RMF in comparison to the culture with antimicrobial not exposed to RMF and expressed as a mean ± SEM calculated from the four repetitions of the experiment; OCT – Octenisept; CHX – Chlorhexidine; Eth.Lac. – Ethacridine lactate; PHMB – Polyhexamethylene biguanide.

| **a** | **b** |
| --- | --- |
| 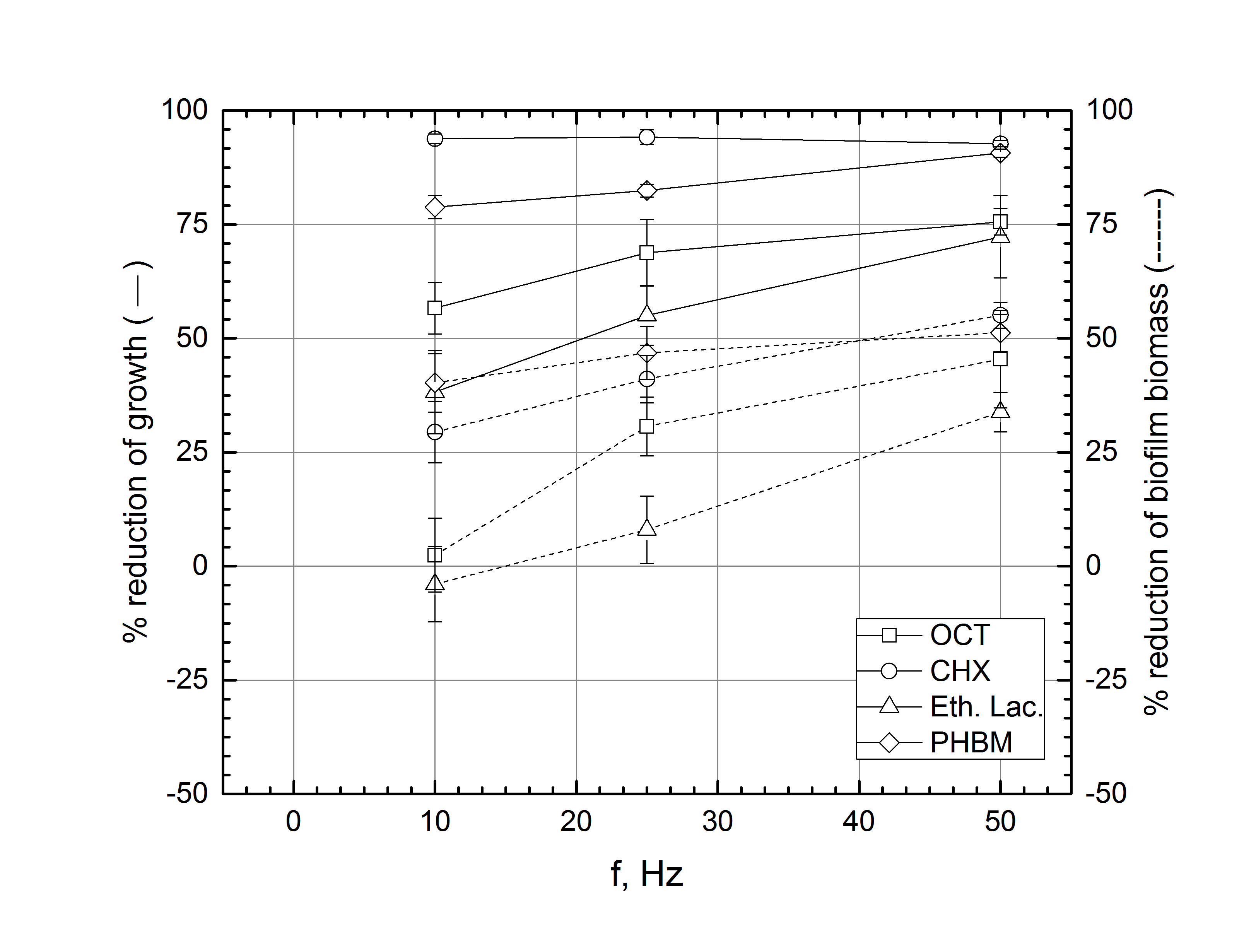 | 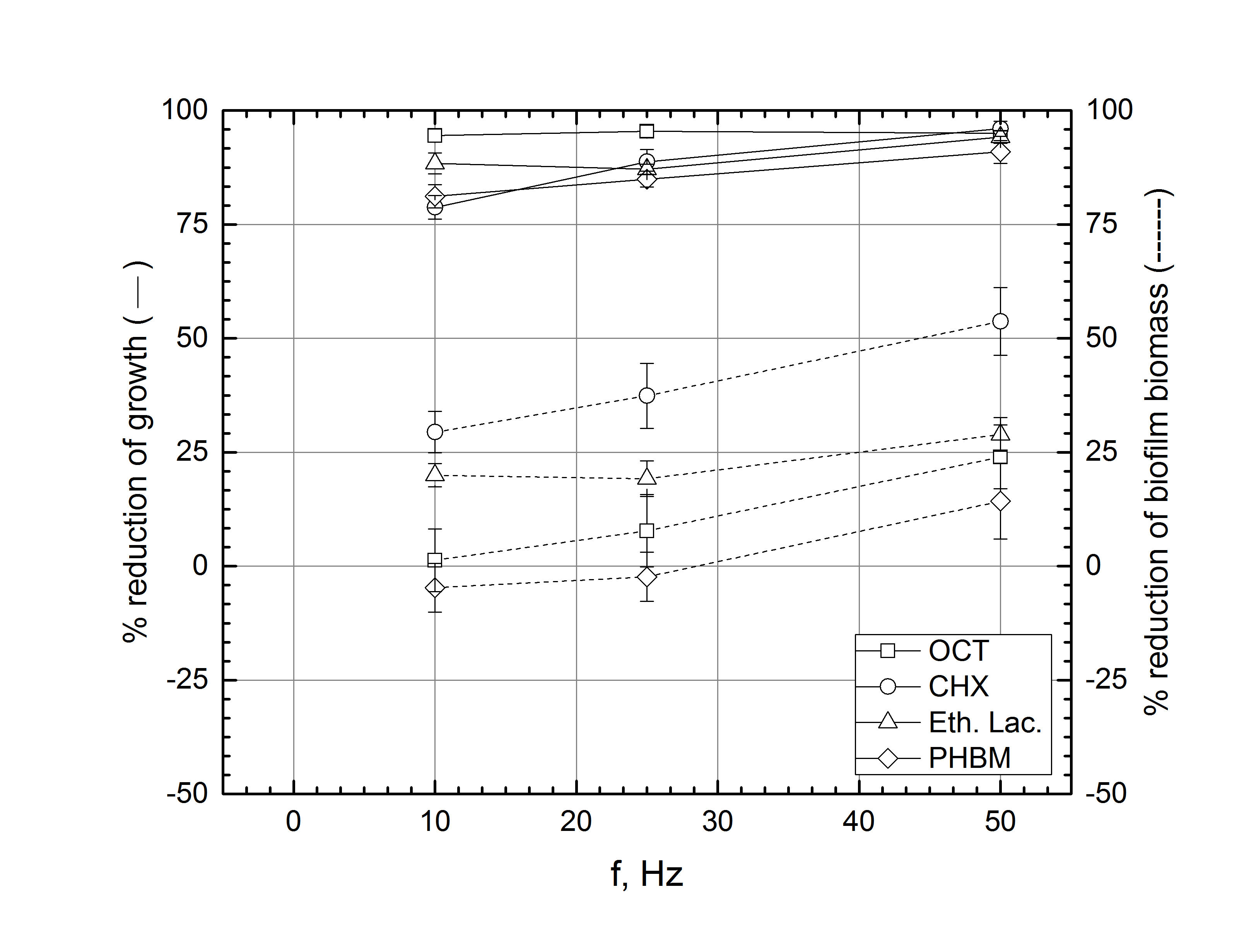 |

**Fig. S5.** Reduction [%] of growth and biofilm biomass of (a) *P. aeruginosa* and (b) *S. aureus* in cultures subjected to RMF for 1 h then incubated for 5 min in medium supplemented with antiseptics.

The results are presented as % reduction of growth and biofilm biomass in cultures subjected to RMF for 1 h then incubated for 5 min in medium supplemented with antimicrobial in comparison to the culture with antimicrobial not exposed to RMF and expressed as a mean ± SEM calculated from the four repetitions of the experiment; OCT – Octenisept; CHX – Chlorhexidine; Eth.Lac. – Ethacridine lactate; PHMB – Polyhexamethylene biguanide.

**Table S1.** Statistically significant differences between means of reduction [%] of biofilm biomass of *P. aeruginosa* and *S. aureus* **exposed to RMF of different frequency in cultures supplemented with antimicrobials.**

| Antimicrobial | RMF frequency (Hz) | | | | | | | | | | | | | | | |
| --- | --- | --- | --- | --- | --- | --- | --- | --- | --- | --- | --- | --- | --- | --- | --- | --- |
|  | 0 (without RMF) | | | | 10 | | | | 25 | | | | 50 | | | |
|  | **a** | | | | **b** | | | | **c** | | | | **d** | | | |
|  | EXP I | | EXP II | | EXP I | | EXP II | | EXP I | | EXP II | | EXP I | | EXP II | |
|  | *P.aeruginosa* | *S.aureus* | *P.aeruginosa* | *S.aureus* | *P.aeruginosa* | *S.aureus* | *P.aeruginosa* | *S.aureus* | *P.aeruginosa* | *S.aureus* | *P.aeruginosa* | *S.aureus* | *P.aeruginosa* | *S.aureus* | *P.aeruginosa* | *S.aureus* |
| Gentamicin | b, c, d | d | b, c, d | d | a, c, d | d | a, c, d | d | a, b, d | d | a, b, d | d | a, b, c | a, b, c | a, b, c | a, b, c |
| Ciprofloxacin | b, c, d | b, c, d | b, c, d | b, c, d | a | a, d | a | a | a | a | a | a | a | a, b | a | a |
| OCT | d | d | c, d | d | d | d | c, d | d | d | d | a, b, d |  | a, b, c | a, b, c | a, b, c | a, b |
| CHX | b, d | b, d | b, c, d | b, c, d | a, d | a, c, d | a, d | a, d | d | b, d | a, d | a, d | a, b, c | a, b, c | a, b, c | a, b, c |
| Eth.Lac. | c, d | b, c, d | d | b, c, d | c, d | a, c, d | d | a | a, b, c | a, b | d | a | a, b, c | a, b | a, b, c | a |
| PHMB | b, c, d | b, c, d | b, c, d | d | a, d | a, c, d | a | d | a | a, b, d | a | d | a, b | a, b, c | a | a, b, c |

The compared results were expressed as % reduction of biofilm biomass of *P. aeruginosa* and *S.* in cultures with antimicrobial in comparison to the culture without antimicrobial after exposure to RMF.

Experimental setting I, antibiotics: exposition of biofilm with gentamicin or ciprofloxacin-supplemented medium to RMF of 10-50 Hz for 1 h.

Experimental setting I, antiseptics: exposition of biofilm with antiseptic to RMF of 10-50 Hz for 5 min.

Experimental setting II, antibiotics: biofilm subjected to RMF of 10-50 Hz for 1 h then incubated for 17 h in medium supplemented with antibiotic.

Experimental setting II, antiseptics: biofilm subjected to RMF of 10-50 Hz for 1 h then incubated for 5 min in antiseptic.

The comparison of % reduction of biofilm biomass of microorganisms depending on RMF frequency and type of antimicrobial were determined by a two-way analysis of variance (ANOVA) and followed by Tukey’s HSD test for post-hoc comparisons. Each experiment was conducted in technical triplicates and repeated four times. Small letters present differences considered as significant at a level of p<0.05. OCT – octenisept; CHX – chlorhexidine; Eth.Lac. – ethacridine lactate; PHMB – polyhexamethylene biguanide.

**Table S2.** Statistically significant differences between means of reduction [%] of growth of *P. aeruginosa* and *S. aureus* **exposed to RMF of different frequency in cultures supplemented with antimicrobials**.

| Antimicrobial | RMF frequency (Hz) | | | | | | | | | | | | | | | |
| --- | --- | --- | --- | --- | --- | --- | --- | --- | --- | --- | --- | --- | --- | --- | --- | --- |
|  | 0 (without RMF) | | | | 10 | | | | 25 | | | | 50 | | | |
|  | **a** | | | | **b** | | | | **c** | | | | **d** | | | |
|  | EXP I | | EXP II | | EXP I | | EXP II | | EXP I | | EXP II | | EXP I | | EXP II | |
|  | *P.aeruginosa* | *S.aureus* | *P.aeruginosa* | *S.aureus* | *P.aeruginosa* | *S.aureus* | *P.aeruginosa* | *S.aureus* | *P.aeruginosa* | *S.aureus* | *P.aeruginosa* | *S.aureus* | *P.aeruginosa* | *S.aureus* | *P.aeruginosa* | *S.aureus* |
| Gentamicin | c, d | b, c, d | b, c, d | b, c, d | d | a, c, d | a | a, d | a, d | a, b | a, d | a | a, b | a, b | a, c | a, b |
| Ciprofloxacin | c, d | b, c, d | b, c, d | b, c, d | d | a, c, d | a, d | a, d | a, d | a, b | a, d | a, b | a, b, c | a, b | a, b, c | a, b |
| OCT | b, c, d | b, c, d | b, c, d | b, c, d | a | a | a, c, d | a | a | a | a, b | a | a | a | a, b | a |
| CHX | b, c, d | b, c, d | b, c, d | b, c, d | a | a | a | a, c, d | a | a | a | a, b, d | a | a | a | a, b, c |
| Eth.Lac. | b, c, d | b, c, d | b, c, d | b, c, d | a, d | a | a, c, d | a | a, d | a | a, b, d | a, d | a, b, c | a | a, b, c | a, c |
| PHMB | b, c, d | b, c, d | b, c, d | b, c, d | a, d | a | a, c, d | a, d | a, d | a | a, b, d | a | a, b, c | a | a, b, c | a, b |

The compared results were expressed as % reduction of growth of *P. aeruginosa* and *S.* in cultures with antimicrobial in comparison to the culture without antimicrobial after exposure to RMF.

Experimental setting I, antibiotics: exposition of biofilm with gentamicin or ciprofloxacin-supplemented medium to RMF of 10-50 Hz for 1 h.

Experimental setting I, antiseptics: exposition of biofilm with antiseptic to RMF of 10-50 Hz for 5 min.

Experimental setting II, antibiotics: biofilm subjected to RMF of 10-50 Hz for 1 h then incubated for 17 h in medium supplemented with antibiotic.

Experimental setting II, antiseptics: biofilm subjected to RMF of 10-50 Hz for 1 h then incubated for 5 min in antiseptic.

The comparison of % reduction of growth of microorganisms depending on RMF frequency and type of antimicrobial were determined by a two-way analysis of variance (ANOVA) and followed by Tukey’s HSD test for post-hoc comparisons. Each experiment was conducted in technical triplicates and repeated four times. Small letters present differences considered as significant at a level of p<0.05. OCT – octenisept; CHX – chlorhexidine; Eth.Lac. – ethacridine lactate; PHMB – polyhexamethylene biguanide.

**Table S3.** Statistically significant differences between means of reduction [%] biofilm biomass of *P. aeruginosa* and *S. aureus* **exposed to RMF of different frequency in cultures supplemented with antimicrobials**.

| Antimicrobial | RMF frequency (Hz) | | | | | | | | | | | |
| --- | --- | --- | --- | --- | --- | --- | --- | --- | --- | --- | --- | --- |
|  | 10 | | | | 25 | | | | 50 | | | |
|  | **a** | | | | **b** | | | | **c** | | | |
|  | EXP I | | EXP II | | EXP I | | EXP II | | EXP I | | EXP II | |
|  | *P.aeruginosa* | *S.aureus* | *P.aeruginosa* | *S.aureus* | *P.aeruginosa* | *S.aureus* | *P.aeruginosa* | *S.aureus* | *P.aeruginosa* | *S.aureus* | *P.aeruginosa* | *S.aureus* |
| Gentamicin | b, c | c | c | c | a, c | c | c | ns | a, b | a, b | a, b | a |
| Ciprofloxacin | b, c | c | ns | ns | a, c | c | ns | ns | a, b | a, b | ns | ns |
| OCT | ns | c | ns | c | ns | c | ns | c | ns | a, b | ns | a, b |
| CHX | ns | b, c | b, c | c | ns | a, c | a, c | c | ns | a, b | a, b | a, b |
| Eth.Lac. | ns | b, c | ns | ns | ns | a | c | ns | ns | a | b | ns |
| PHMB | ns | b, c | c | c | ns | a, c | ns | c | ns | a, b | a | a, b |

The compared results were expressed as % reduction of biofilm biomass of *P. aeruginosa* and *S. aureus* in cultures in medium supplemented with antimicrobial subjected to RMF in comparison to the culture with antimicrobial not exposed to RMF.

Experimental setting I, antibiotics: exposition of biofilm with gentamicin or ciprofloxacin-supplemented medium to RMF of 10-50 Hz for 1 h.

Experimental setting I, antiseptics: exposition of biofilm with antiseptic to RMF of 10-50 Hz for 5 min.

Experimental setting II, antibiotics: biofilm subjected to RMF of 10-50 Hz for 1 h then incubated for 17 h in medium supplemented with antibiotic.

Experimental setting II, antiseptics: biofilm subjected to RMF of 10-50 Hz for 1 h then incubated for 5 min in antiseptic.

The comparison of % reduction of biofilm biomass of microorganisms depending on RMF frequency and type of antimicrobial were determined by a two-way analysis of variance (ANOVA) and followed by Tukey’s HSD test for post-hoc comparisons. Each experiment was conducted in technical triplicates and repeated four times. Small letters present differences considered as significant at a level of p<0.05; ns stands for not statistically significant. OCT – octenisept; CHX – chlorhexidine; Eth.Lac. – ethacridine lactate; PHMB – polyhexamethylene biguanide.

**Table S4.** Statistically significant differences between means of reduction [%] of growth of *P. aeruginosa* and *S. aureus* **exposed to RMF of different frequency in cultures supplemented with antimicrobials**.

| Antimicrobial | RMF frequency (Hz) | | | | | | | | | | | |
| --- | --- | --- | --- | --- | --- | --- | --- | --- | --- | --- | --- | --- |
|  | 10 | | | | 25 | | | | 50 | | | |
|  | **a** | | | | **b** | | | | **c** | | | |
|  | EXP I | | EXP II | | EXP I | | EXP II | | EXP I | | EXP II | |
|  | *P.aeruginosa* | *S.aureus* | *P.aeruginosa* | *S.aureus* | *P.aeruginosa* | *S.aureus* | *P.aeruginosa* | *S.aureus* | *P.aeruginosa* | *S.aureus* | *P.aeruginosa* | *S.aureus* |
| Gentamicin | b, c | b, c | b, c | b, c | a, c | a | a | a | a, b | a | a | a |
| Ciprofloxacin | ns | c | ns | c | c | ns | c | ns | b | a | b | a |
| OCT | ns | c | c | b, c | ns | c | ns | a, c | ns | a, b | a | a, b |
| CHX | ns | c | ns | c | ns | c | ns | c | ns | a, b | ns | a, b |
| Eth.Lac. | c | b, c | b, c | c | c | a, c | a, c | c | a, b | a, b | a, b | a, b |
| PHMB | ns | c | ns | ns | ns | ns | ns | ns | ns | a | ns | ns |

The compared results were expressed as % reduction of growth of *P. aeruginosa* and *S. aureus* in cultures in medium supplemented with antimicrobial subjected to RMF in comparison to the culture with antimicrobial not exposed to RMF.

Experimental setting I, antibiotics: exposition of biofilm with gentamicin or ciprofloxacin-supplemented medium to RMF of 10-50 Hz for 1 h.

Experimental setting I, antiseptics: exposition of biofilm with antiseptic to RMF of 10-50 Hz for 5 min.

Experimental setting II, antibiotics: biofilm subjected to RMF of 10-50 Hz for 1 h then incubated for 17 h in medium supplemented with antibiotic.

Experimental setting II, antiseptics: biofilm subjected to RMF of 10-50 Hz for 1 h then incubated for 5 min in antiseptic.

The comparison of % reduction of growth of microorganisms depending on RMF frequency and type of antimicrobial were determined by a two-way analysis of variance (ANOVA) and followed by Tukey’s HSD test for post-hoc comparisons. Each experiment was conducted in technical triplicates and repeated four times. Small letters present differences considered as significant at a level of p<0.05; ns stands for not statistically significant. OCT – octenisept; CHX – chlorhexidine; Eth.Lac. – ethacridine lactate; PHMB – polyhexamethylene biguanide.
